# USP35, regulated by estrogen and AKT, promotes breast tumorigenesis by stabilizing and enhancing transcriptional activity of estrogen receptor α

**DOI:** 10.1038/s41419-021-03904-4

**Published:** 2021-06-15

**Authors:** Jiawei Cao, Du Wu, Guang Wu, Yaqi Wang, Tianhao Ren, Yang Wang, Yingshuai Lv, Wei Sun, Jieyi Wang, Changrui Qian, Licai He, Kaiyan Yang, Hongzhi Li, Haihua Gu

**Affiliations:** 1 Key Laboratory of Laboratory Medicine, Ministry of Education, School of Laboratory Medicine and Life Sciences, Wenzhou Medical University, Wenzhou, 325035 China; 2 Department of Pathology, The First Affiliated Hospital of Wenzhou Medical University, Wenzhou, China

**Keywords:** Breast cancer, Tumour biomarkers, Cell growth

## Abstract

Although endocrine therapies targeting estrogen receptor α (ERα) are effective in managing ER positive (+) breast cancer, many patients have primary resistance or develop resistance to endocrine therapies. In addition, ER+ breast cancer with *PIK3CA* activating mutations and 11q13-14 amplification have poor survival with unclear mechanism. We uncovered that higher expression of deubiquitinase *USP35*, located in 11q14.1, was associated with ER+ breast cancer and poor survival. Estrogen enhanced USP35 protein levels by downregulating USP35-targeting miRNA-140-3p and miRNA-26a-5p. USP35 promoted the growth of ER+ breast cancer in vitro and in vivo, and reduced the sensitivity of ER+ breast cancer cells to endocrine therapies such as tamoxifen and fulvestrant. Mechanistically, USP35 enhanced ERα stability by interacting and deubiquitinating ERα, and transcriptional activity of ERα by interacting with ERα in DNA regions containing estrogen response element. In addition, AKT, a key effector of PI3K, phosphorylated USP35 at Serine613, which promoted USP35 nuclear translocation, ERα transcriptional activity, and the growth of ER+ breast cancer cells. Our data indicate that USP35 and ERα form a positive feedback loop in promoting the growth of ER+ breast cancer. USP35 may be a treatment target for ER+ breast cancer with endocrine resistance or with *PIK3CA* mutations or hyperactivation of the PI3K pathway.

## Introduction

Up to 70% of breast cancer is driven by estrogen receptor α (ERα) [1]. Upon binding to estrogen, ERα translocates into the nucleus, binds DNA regions containing estrogen-responsive element (ERE), and regulates the transcription of a plethora of genes important for breast tumorigenesis [2, 3]. Anti-estrogen-based endocrine therapies significantly improve survival of ERα positive (ER+) breast cancer [4]. However, about half of the patients treated with endocrine therapies endure relapse, making it a significant clinical problem [5].

Various mechanisms account for the endocrine resistance in ER+ breast cancer, including mutations in ERα gene (*ESR1*), ERα post-translation modifications, and activation of the phosphatidylinositol 3-kinase (PI3K) pathway or *PIK3CA* activating mutations [6, 7]. Approximately 40% of the patients with hormone receptor-positive, HER2-negative breast cancer have activating mutations in *PIK3CA*, which encodes the catalytic subunit of PI3K, p110α, displaying hyperactivity [8, 9]. PI3K-p110α specific inhibitor alpelisib significantly improved the efficacy of endocrine therapy against previously treated ER+ breast cancer with *PIK3CA* mutations [10]. Although breast cancer patients with *PIK3CA* mutation harboring primary tumors have prolonged overall survival [9, 11], a recent study revealed that ER+ breast cancer with *PIK3CA* mutations and reduced survival are associated with three subgroups with amplification at the 17q23, 11q13-14, or 8q24 loci respectively [12].

Ubiquitination affects the stability and transcriptional activity of ERα. Protein ubiquitination is controlled by the balanced activities of ubiquitin ligase and deubiquitinase [13]. More is known about the ubiquitin ligases that ubiquitinate ERα. For example, CHIP, BRCA1, and Hbo1 had been reported to destabilize ERα and inhibit ERα transcriptional activity by polyubiquitinating ERα [14–16]. RNF31 promoted ERα protein stability and enhances ERα signaling by eliciting mono-ubiquitination [17]. However, less is known about the cellular factors that trigger ERα deubiquitination.

*USP35*, encoding Ubiquitin Specific Peptidase 35 (USP35), is located on chromosome *11q14.1*, where a small amplicon including the four genes *GAB2, USP35, KCTD21, and ALG8* was amplified around 9% in breast cancer patients (www.cbioportal.org) [18]. We and others have showed that Gab2 overexpression promotes breast tumorigenesis and metastasis through activation of the PI3K and Shp-2/Erk pathways [19–21]. However, the role of USP35 in breast cancer is still unknown.

Four different isoforms of USP35 have been reported so far [22, 23]. The full length isoform1 consists of an N-terminal HEAT domain and the C-terminal USP catalytic domain. It is mainly present in cytoplasm, functioning as an anti-apoptotic protein. Isoform2, which contains only part of the HEAT domain, is located in the endoplasmic reticulum. Isoform2 overexpression caused rapid endoplasmic reticulum stress [23]. Isoform3 lacks the HEAT domain with an unknown function. Another shorter form of USP35 (s-USP35) has been reported to delay PARK2/Parkin-mediated mitophagy through transient association with polarized mitochondria [22]. In addition, USP35 is involved in regulating mitotic progression by maintaining the stability of Aurora B through deubiquitination during mitosis [24]. The role of USP35 in cancer is less well understood. One report suggested that USP35 acts as a tumor suppressor [25]. In contrast, a recent study revealed that USP35 is overexpressed in ovarian cancer with poor prognosis. USP35 via deubiquitination and inactivation of STING restrains the activation of the STING-TBK1-IRF3 pathway and type I interferon production, important for enhancing anti-tumor immunity, in ovarian cancer cells [26].

In the current study, we uncovered that higher USP35 expression is associated with ER+ breast cancer and poor prognosis. Importantly, USP35 promotes the growth of ER+ breast cancer in vitro and in vivo, and reduces the response to endocrine therapies by enhancing the stability and transcriptional activity of ERα. Furthermore, phosphorylation of Ser613 in USP35 by AKT, an effector of PI3K, is critical for USP35 nuclear translocation and promoting ERα transcriptional activity and the growth of ER+ breast cancer cells.

## Results

### USP35 is significantly overexpressed in ER+breast cancer and predicts poor outcome

**Fig. 1:**
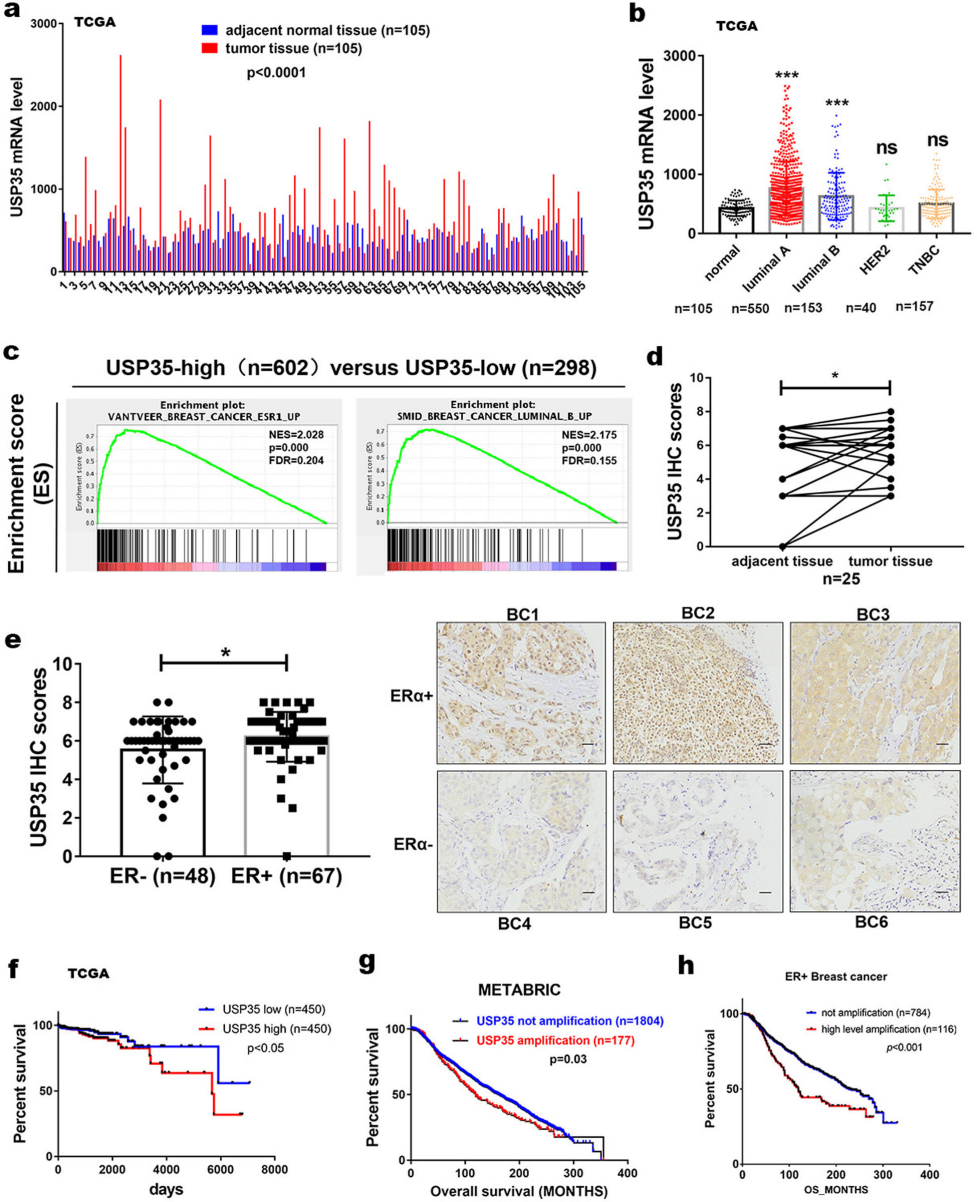
Higher USP35 expression is associated with ER+ breast cancer and predicts poor outcome. **a** USP35 mRNA levels in tumor tissues are significantly higher than in normal tissues. *p* < 0.0001, two-tailed paired Student’s *t*-test. **b** USP35 mRNA level is higher in luminal than in other subtypes of breast cancer. **, *p* < 0.01; ***, *p* < 0.001; ns not significant, one-way ANOVA. **c** Gene Enrichment Set Analysis (GESA) showed that breast cancer with high USP35 mRNA level was enriched with the ESR1 up, luminal B up sets. **d** USP35 immunohistochemistry analysis showed that USP35 protein levels were higher in primary breast tumor tissues than in paired adjacent normal tissues. **e** Immunohistochemistry analysis showed that USP35 protein levels were significantly higher in ER+ than in ER− breast tumors (left). *, *p* < 0.05, two-tailed Student’s *t*-test. USP35 staining in different ER+ and ER− breast tumors were shown (right). Scale bar, 100 μm. Kaplan–Meier analysis for overall survival of breast cancer patients depending on USP35 mRNA levels in the TCGA database (**f**), USP35 amplification status in breast cancer (**g**), and in ER+ breast cancer (**h**) in the METABRIC database were shown. *p* value was calculated using the log-rank test.

Because an amplicon in 11q14.1 containing *USP35* was found in a small subset of breast cancer, we first analyzed *USP35* expression in breast cancer cohort in publicly available databases, such as TCGA and METABRIC. *USP35* mRNA was overexpressed in primary breast carcinomas compared with adjacent normal tissues (Fig. 1a). Importantly, *USP35* mRNA levels were higher in luminal (ER+) than in other subtypes of breast cancer (Fig. 1b). In addition, Gene Set Enrichment Analysis (GSEA) also revealed that breast cancer with *USP35*-high expression was enriched in *ESR1* up and luminal B up breast cancer (Fig. 1c). These observations were supported by immunohistochemistry analysis of USP35 in primary breast tumor specimens. USP35 protein level was significantly higher in tumors than in adjacent normal tissues (Fig. 1d). Importantly, USP35 protein expression was higher in ER+ than in ER negative (−) tissues (Fig. 1e). Interestingly, USP35 protein was predominately localized in the nucleus of some ER+ breast tumor samples (Fig. 1e, BC1 and BC2), and in the cytosol and nucleus of other samples (Fig. 1e, BC3). Furthermore, Kaplan–Meier analysis showed that breast cancer with higher *USP35* mRNA level (Fig. 1f) exhibited significantly shorter overall survival time than those with lower mRNA level (Fig. 1f). Similarly, the overall survival time of breast cancer with *USP35* amplification (8.9%) or ER+ breast cancer with *USP35* amplification (12.9%) was shorter than those without amplification (Fig. 1g). These results indicate that higher USP35 status is associated with ER+ breast cancer and is a poor prognostic marker for breast cancer.

### Estrogen promotes USP35 expression by downregulating miR-140-3p and miR-26a-5p in ER+ breast cancer cells

**Fig. 2:**
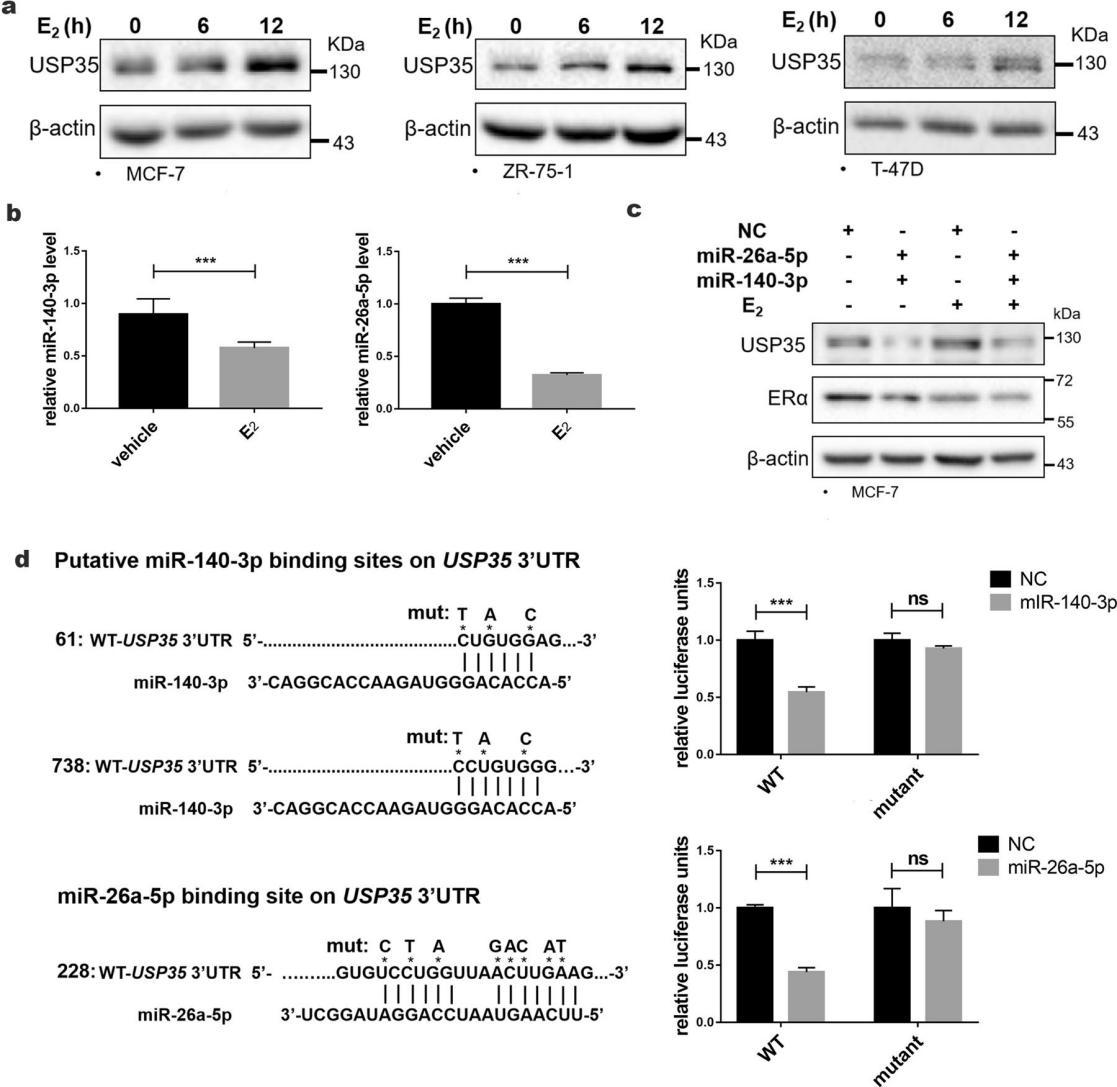
Estrogen increases USP35 expression via decreasing miR-140-3p and miR-26a-5p levels in ER + breast cancer cells. **a** E_2_ increases USP35 protein level. MCF-7, ZR-75-1, and T-47D cells were treated with 10 nM E_2_ and immunoblotted with the indicated antibodies. **b** E_2_ decreases miR-26a-5p and miR-140-3p levels. MCF-7 cells were treated with vehicle or E_2_ (10 nM) and subjected to qRT-PCR analysis. ***, *p* < 0.001. **c** miR-140-3p and miR-26a-5p can reduce E_2_-enhanced USP35 protein levels. MCF-7 cells were transfected with scramble negative control RNA (NC), the indicated miRNA mimics, respectively, and treated with E_2_ for 24 h before being subjected to immunoblotting. **d** *USP35* 3′UTR contains the predicted miR-140-3p target sites in positions 61–66 and 738–744 (top left) and miR-26a-5p target site in position 228–234 (bottom left). MCF-7 cells were co-transfected with NC, miR-140-3p, or miR-26a-5p mimics respectively, together with *USP35*-3′UTR-WT-luc or *USP35*-3′UTR-mut-luc plasmids. The top and bottom right panels contain the *USP35*-3′UTR-mut-luc plasmids in which the miR-140-3p and miR-26a-5p target sites were mutated respectively. The normalized relative luciferase activities were shown on the right panels. ns not significant; ***, *p* < 0.001.

Because higher USP35 expression is associated with ER+ breast cancer, we tested the hypothesis that ERα may be involved in regulating USP35 expression. Immunoblotting analysis revealed that USP35 protein level was enhanced significantly after estradiol (E_2_) treatment in three different ER+ breast cancer cell lines (Fig. 2a). miRNAs regulates protein expression by targeting *3*′*-UTR* in mRNAs [27]. miR-26a-5p and miR-140-3p were reported to be downregulated by estradiol treatment [28–30]. Examination of *USP35* mRNA sequence using Targetscan site (http://www.targetscan.org) revealed that *USP35-3*′*UTR* contains two predicted miR-140-3p binding sites and one predicted miR-26a-5p binding site (Fig. 2d). Importantly, estradiol treatment inhibited miR-26a-5p and miR-140-3p expression in MCF-7 cells (Fig. 2b), whereas mRNA levels of ERα target genes (*MYC* and *CCND1*) were increased (Fig. S2). Furthermore, miR-140-3p or miR-26a-5p mimic reduced USP35 protein levels in MCF-7 cells (data now shown). Transfection of both miR-140-3p and miR-26a-5p mimics together decreased E_2_-induced USP35 protein levels in comparison with control miRNA mimic (Fig. 2c). To test whether miR-26a-5p and miR-140-3p target *USP35*, luciferase (luc) reporter plasmids containing *USP35 3*′*UTR-*WT or mutant fragments, in which the predicted miR-140-3p and miR-26a-5p binding sites were mutated (Fig. 2d, left panels), were contransfected with miR-140-3p or miR-26a-5p mimics into MCF-7 cells (Fig. 2d). miR-140-3p (Fig. 2d, upper right) or miR-26a-5p (Fig. 2d, lower right) mimic significantly decreased *USP35 3*′*UTR-*WT-luc reporter activities, whereas miR-140-3p or miR-26a-5p mimic had no effect on the activity of the luc-reporter containing its corresponding *USP35-3*′*UTR-*mutant fragment. This result demonstrated that *USP35* is a bona fide target of miR-26a-5p and miR-140-3p. Our data support a model that estradiol promotes USP35 expression by downregulating miR-26a-5p and miR-140-3p in ER+ breast cancer cells.

### USP35 expression promotes the growth of ER+ breast cancer in vitro and in vivo

**Fig. 3:**
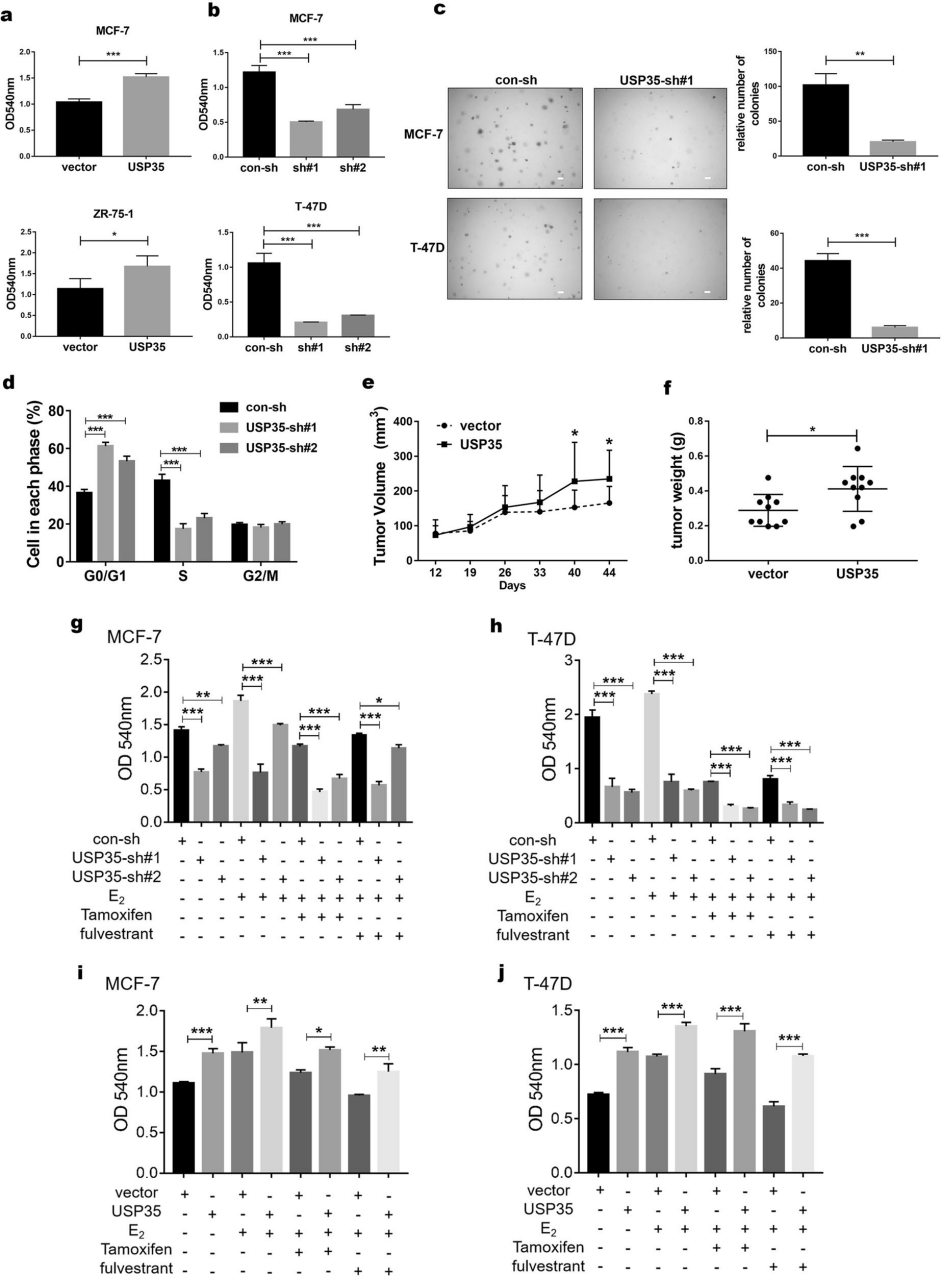
USP35 is critical for the growth of ER+ breast cancer in vitro and in vivo, and decreases the sensitivity of cells to endocrine therapies. **a**, **b** USP35 promotes the growth of ER+ breast cancer cells. MCF-7 and ZR-75-1 cells with USP35 overexpression (**a**), or MCF-7 and T-47D cells with USP35 knockdown (**b**) were subjected to colony formation assays. **c** Knockdown of USP35 inhibits anchorage independent growth of ER+ breast cancer cell. Representative images of cell colonies in soft agar are shown on the left and the quantitated results were shown on the right. Scale bar, 50 μm. **d** Knockdown of USP35 causes cells arrested in G1 phase. DNA content in different cell cycle phase in MCF-7 cells was analyzed by FACS. ***, *p* < 0.001. **e**, **f** USP35 expression promotes the growth of ER+ breast tumors in NCG mice. MCF-7 cells stably expressing vector or USP35 were injected into the mammary fat pad of NCG mice (*n* = 5 per group). Tumor growth (**e**) and weights at week 7 (**f**) were shown. **g**, **h** Knockdown of USP35 increases the sensitivity of ER+ breast cancer cells to Tamoxifen or fulvestrant treatment. MCF-7 cells (**g**) and T-47D cells (**h**) with control-shRNA and USP35-shRNAs (sh#1, sh#2) were hormone-starved for 3 d and then treated with 10 nM E_2_, together without and with 5 μM Tamoxifen or 1 μM fulvestrant for 3 d before being subjected to colony formation assays. **i**, **j** USP35 overexpression decreases the sensitivity of ER+ breast cancer cells to Tamoxifen or fulvestrant treatment. MCF-7 (**i**) and T-47D (**j**) cells with vector or USP35 overexpression were treated and assayed as described above (**g**, **h**). *, *p* < 0.05; **, *p* < 0.01; ***, *p* < 0.001.

To explore the function of USP35 in ER+ breast cancer, we first examined USP35 protein levels in different types of breast cancer cell lines. USP35 protein levels were higher in commonly used ER+ (i.e., MCF-7, ZR-75-1, T-47D) than ER− breast cancer cell lines (Fig. S1a). Next, USP35 was overexpressed (Fig. S1b) and knocked down using two different *USP35* shRNAs (Fig. S1c) in these ER+ breast cancer cells. USP35 overexpression promoted the growth of cells (Fig. 3a), whereas USP35 knockdown markedly reduced cell growth in colony formation (Fig. 3b) and soft agar (Fig. 3c) assays. Flow cytometry analysis revealed that USP35 knockdown increased cell content in G1 phase and reduced cell content in S phase of the cell cycle (Fig. 3d). To further test the role of USP35 in vivo, MCF-7 cells stably expressing vector and USP35 were injected into the 4th mammary fat pads of the immunodeficient mice. USP35 overexpression enhanced the growth of MCF-7 tumors compared with vector control (Figs. 3e and 3f). These results indicate that USP35 acts as an oncogene in ER+ breast cancer.

### USP35 expression promotes resistance to endocrine therapies

Given that USP35 expression is associated with ER+ breast cancer, we further investigated whether changes in USP35 levels affect the response of ER+ breast cancer cells to the commonly used drugs in endocrine therapies. As expected, tamoxifen or fulvestrant treatment inhibited the E_2_-stimulated growth of MCF-7 and T-47D cells (Fig. 3g–j). Knockdown of USP35 enhanced the growth inhibition of MCF-7 by Tamoxifen or fulvestrant (Fig. 3g), whereas overexpression of USP35 negated the growth inhibition of MCF7 cells by tamoxifen or fulvestrant (Fig. 3h). Likewise, knockdown of USP35 also increased (Fig. 3i), whereas USP35 overexpression reduced (Fig. 3j) the growth inhibition of T-47D cells by tamoxifen or fulvestrant. USP35 protein levels in cells from Figs. 3g–3j were shown in Fig. S3. These results show that higher USP35 expression contributes to endocrine resistance of ER+ breast cancer cells.

### USP35 regulates ERα protein level by interacting with and deubiquitinating ERα

**Fig. 4:**
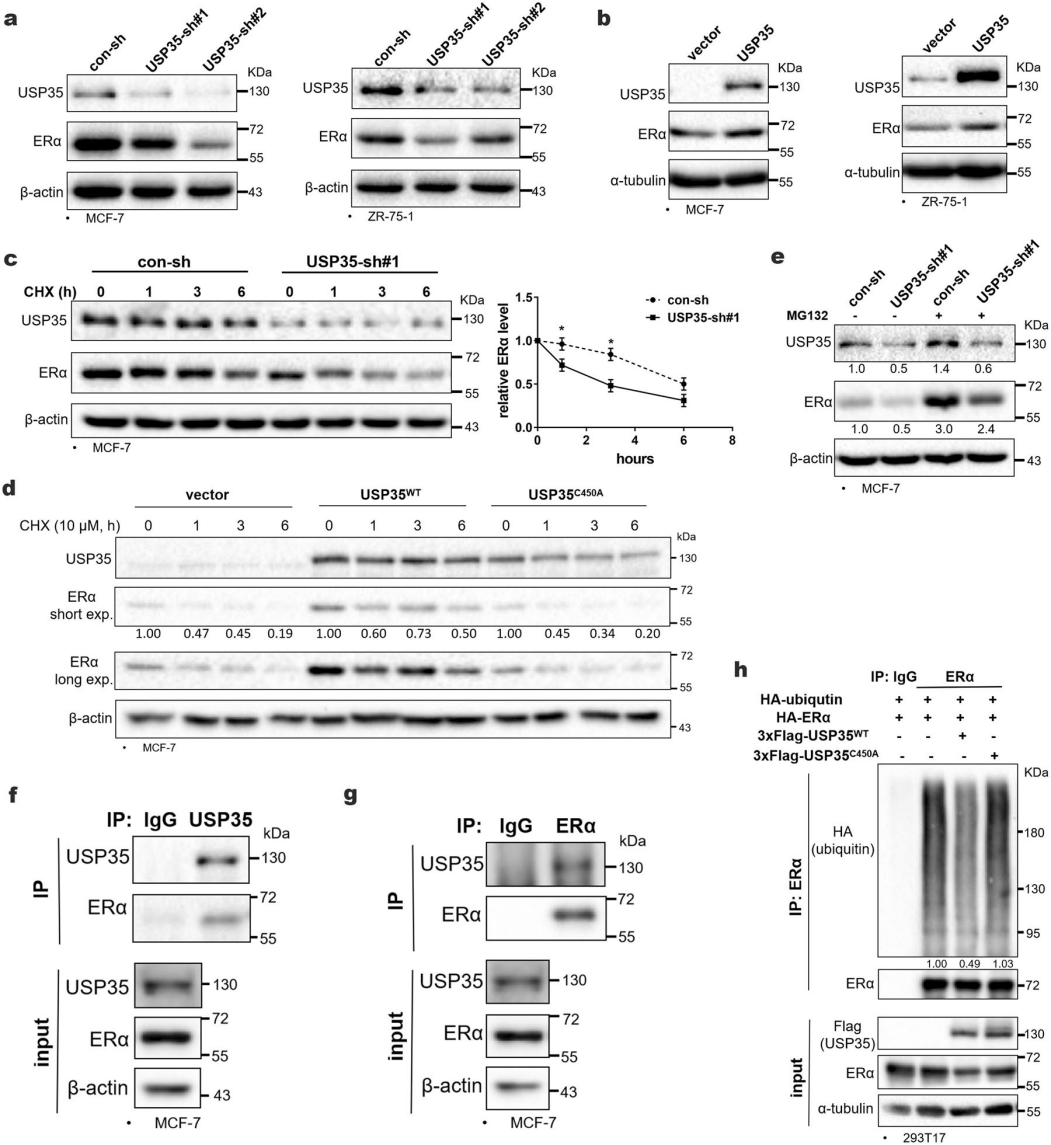
USP35 regulates ERα protein level by interacting and deubiquitinating ERα. Knockdown of USP35 inhibits (**a**) and USP35 overexpression enhances (**b**) ERα protein level in ER+ breast cancer cells. **c**, **d** USP35 promotes ERα protein stability. MCF-7 cells infected with lentivirus expressing con-sh and USP35-sh#1 (**c**), and MCF-7 cells stably expressing vector, USP35^WT^, and USP35^C450A^ (**d**) were treated in the presence of cycloheximide (CHX, 10 μM) and E_2_ (10 nM) for the indicated times. **e** MG132 reverses decreased ERα expression caused by USP35 knockdown. MCF-7 cells with con-sh or USP35-sh#1 were treated with MG132 (10 μM) for 6 h. **f**, **g** USP35 interacts with ERα in breast cancer cells. MCF-7 cell lysates were immunoprecipitated with anti-USP35 antibodies and rabbit IgG (negative control) (**f**), or anti-ERα antibody and mouse IgG (negative control) (**g**), followed by immunoblotting with the indicated antibodies. **h** USP35 promotes ERα deubiquitination. 293T17 cells were cotransfected with HA-ubiquitin and HA-ERα together with 3 x Flag-USP35^WT^ or 3 x Flag-USP35^C450A^ plasmids, and treated with MG132 (10 μM) for 6 h. Cell lysates were immunoprecipitated with anti-ERα antibody and mouse IgG.

Considering the important role of ERα in ER+ breast cancer, we investigated whether USP35 regulates the ERα protein level. Knockdown of USP35 strongly decreased (Fig. 4a), whereas overexpression of USP35 increased (Fig. 4b) ERα protein level in ER+ breast cancer cell lines (MCF-7 and ZR-75-1). To test whether USP35 regulates the stability of ERα protein, cells treated with cycloheximide (a protein synthesis inhibitor) in the presence of E_2_. Knockdown of USP35 accelerated ERα turnover in comparison to control-shRNA (Fig. 4c). Conversely, USP35^WT^ overexpression decreased ERα turnover in comparison to vector control. In contrast, USP35^C450A^, in which the catalytic cysteine was mutated into alanine, failed to promote ERα stability in comparison to USP35^WT^ (Fig. 4d). Furthermore, MG132 (a proteasome inhibitor) treatment prevented the decrease in ERα protein level caused by USP35 knockdown (Fig. 4e).

Since proteasome degrades ubiquitin-tagged proteins, we hypothesized that USP35 may interact with and deubiquitinate ERα. Results from immunoprecipitation experiment in MCF-7 cells revealed that USP35 and ERα can be reciprocally coimmunoprecipitated (Figs. 4f, g), indicating that USP35 interacted with endogenous ERα. In addition, cotransfection experiment in 293T17 also showed that ERα was coimmunoprecipitated with USP35 (Fig. S4a). To test whether USP35 deubiqutinates ERα, HA-ERα, and HA-ubiquitin plasmids were cotransfected with vector expressing USP35^WT^ or USP35^C450A^ into 293 cells. Compared to vector control, USP35^WT^ decreased ubiquitination of ERα (Fig. 4h), whereas USP35^C450A^ did not affect ubiquitination of ERα. In addition, result from HA-ubiquitin immunoprecipitation followed by immunoblotting with the anti-ERα antibodies also showed that USP35^WT^ significantly reduced ubiquitination of ERα (Fig. S4b). Furthermore, knockdown of USP35 also increased ERα ubiquitination in ER+ breast cancer cells (Fig. S4c). These results indicate that USP35 interacts with and deubiquitinates ERα.

### USP35 enhances ERα transcriptional activity by binding to DNA regions containing the estrogen responsive element in ERα target genes

**Fig. 5:**
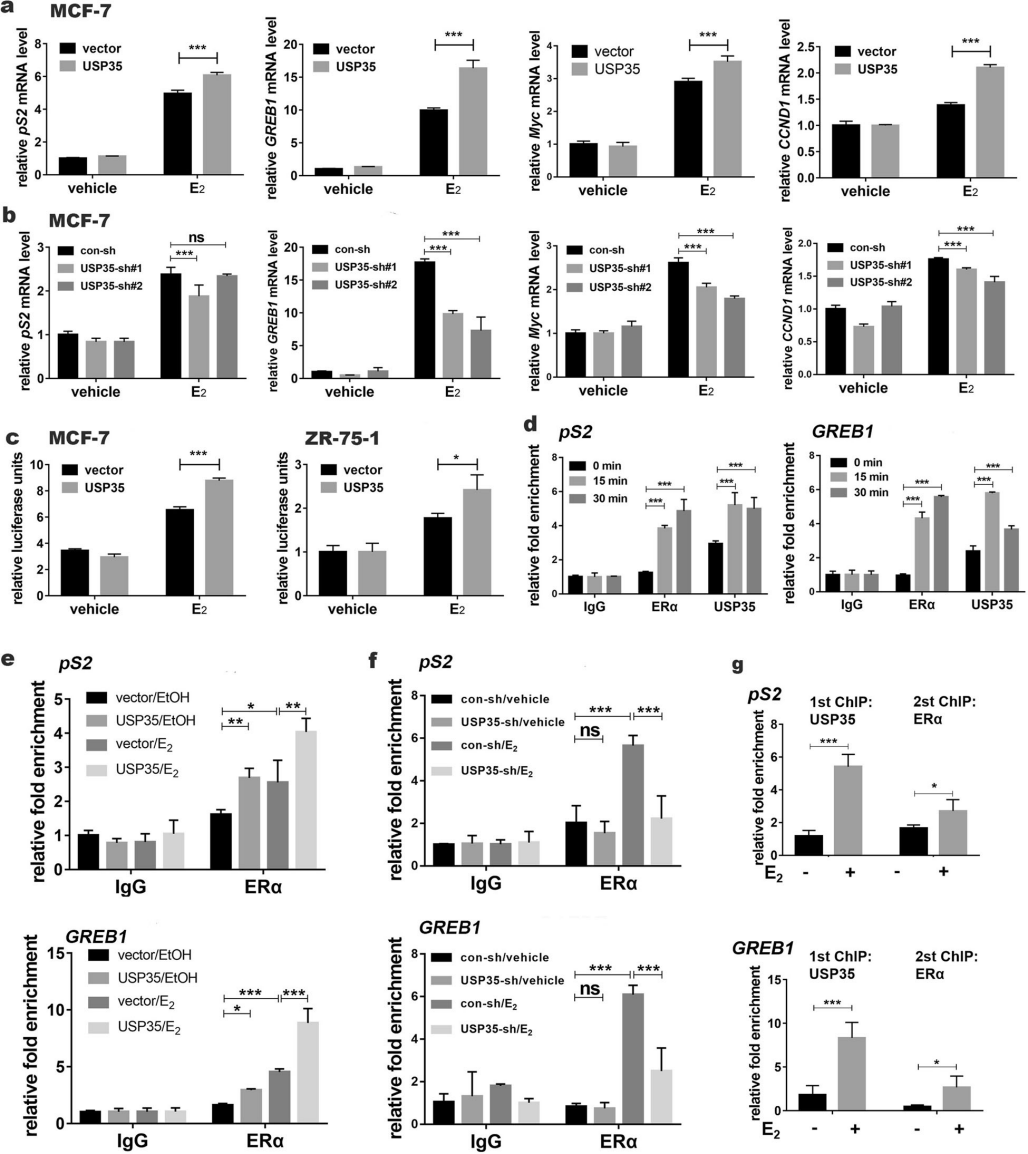
USP35 enhances ERα transcriptional activity by binding to estrogen responsive element containing region in ERα target genes. USP35 overexpression increases (**a**) and USP35 knockdown decreases (**b**) mRNA levels of the estrogen induced genes. Indicated MCF-7 cell lines were treated with vehicle or E_2_ (10 nM) for 6 h before subjected to qRT-PCR assay. **c** USP35 overexpression enhances ERE-luciferase reporter activity. Indicated cell lines were cotransfected with C3-ERE-luc reporter and TK-renilla plasmids, treated with E_2_ (10 nM) for 24 h, and subjected to luciferase activity assay. TK renilla luciferase was used to normalize transfection efficiency. **d** Estrogen enhances USP35 binding to ERE-containing region in the ERα target genes. MCF-7 cells stimulated with E_2_ (10 nM) were subjected to ChIP assay using USP35 or ERα antibody followed by qPCR of the *pS2* and *GREB1* DNA regions containing ERE. The quantification of fold enrichment relative to input levels was shown. **e**, **f** USP35 promotes ERα binding to ERE-containing region in *pS2* and *GREB1* in MCF-7 cells. MCF-7 cells stably expressing vector and USP35 (**e**) or MCF-7 cells expressing con-sh and USP35-sh#1 (**f**) were stimulated with E_2_ (10 nM) for 15 min and subjected to ChIP assay using ERα antibody. **g** USP35 and ERα are recruited together to ERE-containing regions of *pS2* and *GREB1*. MCF7 cells were ChIP and re-ChIP using USP35 and ERα antibodies sequentially. Chromatin samples were analyzed by qPCR. *, *p* < 0.05; ***, *p* < 0.001.

To test whether USP35 affect the expression of ERα target genes, mRNA levels of *pS2, GREB1, Myc, and CCND1* were analyzed in ER+ breast cancer cells with USP35 overexpression (Fig. 5a) or USP35 knockdown (Fig. 5b) by qRT-PCR. E_2_-induced *pS2, GREB1, Myc and CCND1* mRNA levels were enhanced by USP35 overexpression compared with vector control (Fig. 5a), and reduced by USP35 knockdown compared with control-sh (Fig. 5b).

To test whether USP35 also affects ERα transcriptional activity, USP35 expression plasmid was cotransfected with the C3 promoter-luciferase reporter plasmid into MCF-7 and ZR-75-1 cells. Complement C3 promoter contains estrogen response element (ERE) [31]. USP35 overexpression enhanced E_2_-induced luciferase reporter activity in comparison with vector control (Fig. 5c).

Because USP35 was detected in the nucleus of ER+ breast tumor cells (Fig. 1e), we postulated that USP35 affects ERα regulated gene transcription by binding to ERE-containing DNA regions. Chromatin immunoprecipitation (ChIP) assay was used to assess USP35 binding to the ERE-containing regions in *pS2* and *GREB1*. Under E_2_-starved condition, ERα had no basal binding, whereas USP35 showed some basal binding to the ERE-containing regions. E_2_ treatment-induced ERα, and further enhanced USP35 binding to these regions (Fig. 5d). In addition, USP35 overexpression promoted basal and E_2_-stimulated binding of ERα to the ERE-containing regions (Fig. 5e), whereas USP35 knockdown inhibited these bindings (Fig. 5f). We further investigated whether both USP35 and ERα are present together in DNA regions containing ERE. ChIP assays were performed using USP35 antibodies first, eluted and reChIP using ERα antibody for the ERE-containing regions in *pS2* and *GREB1* (Fig. 5g). Our data revealed that USP35 and ERα were recruited together to the same ERE-containing region in ERα target genes upon estrogen stimulation. Taken together, our results indicate that USP35 is recruited to the ERE-containing regions in ERα target genes to modulate ERα transcriptional activity.

### AKT promotes nuclear translocation of USP35 and enhances ERα transcriptional activity by phosphorylating USP35 at Ser613

**Fig. 6:**
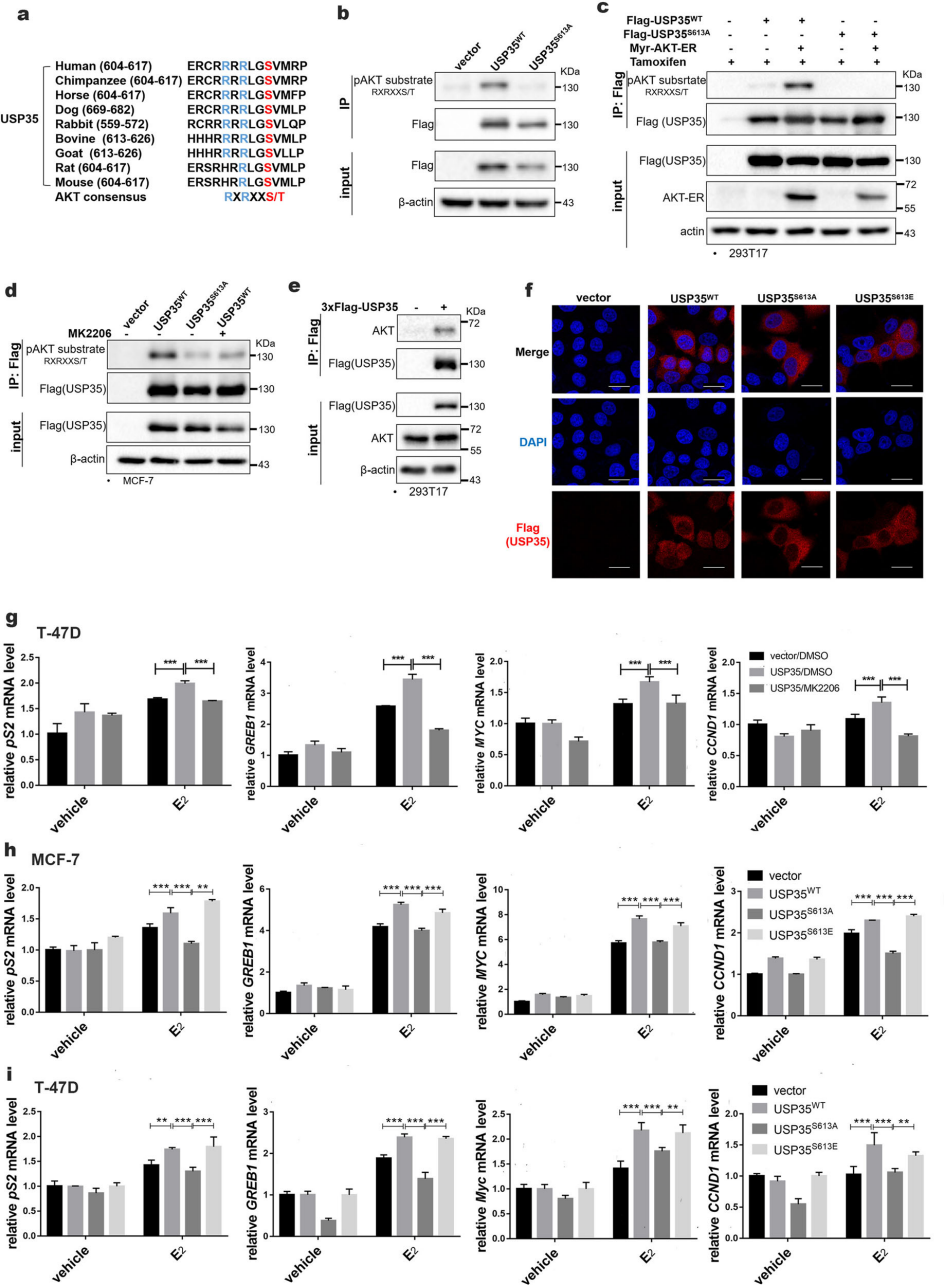
AKT promotes the nuclear translocation of USP35 and enhances ERα transcriptional activity by phosphorylating USP35 at Ser613. **a** Ser613 containing motif in USP35 is a conserved AKT phosphorylation site among mammals except for rats and mice. **b** USP35 is phosphorylated at Ser613 in ER+ breast cancer cells. T47D cells expressing vector, Flag-USP35^WT^, and Flag-USP35^S613A^ were subjected to immunoprecipitation with anti-Flag antibody followed by immunoblotting with phosphor-AKT substrate antibodies. **c** AKT can phosphorylate Ser613 in USP35. 293T17 cells were co-transfected with Flag-USP35^WT^ or Flag-USP35^S613A^ together with myr-AKT-ER (inducible activation of AKT) plasmids and treated with tamoxifen (5 μM) 6 h before being subjected to immunoprecipitation with anti-Flag antibody. **d** AKT inhibitor MK2206 inhibits Ser613 phosphorylation in USP35. Indicated MCF-7 cell lines were treated with vehicle or MK2206 (100 nM) for 1 h and subjected to immunoprecipitation with anti-Flag antibody followed by immunoblotting. **e** AKT interacts with USP35. 293T17 cells were transfected with Flag-USP35 plasmids and subjected to immunoprecipitation using anti-Flag antibody and immunoblotting. **f** Ser613 is critical for nuclear translocation of USP35 in breast cancer cells. Indicated MCF-7 cell lines were immunostained with anti-Flag antibody (red) and DAPI (blue), and examined by confocal microscopy. Bar = 25 μm. **g** AKT inhibitor blocks UPS35-enhanced estrogen regulated gene expression. Indicated T-47D cells were pretreated with DMSO or MK2206 (100 nM) for 1 h, and then treated with vehicle or E_2_ (10 nM) for 6 h. **h**, **i** Ser613 phosphorylation is critical for UPS35-enhanced estrogen-regulated gene expression. MCF-7 (**h**) and T-47D (**i**) cells expressing vector, USP35^WT^, USP35^S613A^, and USP35^S613E^ were treated with vehicle or E_2_ (10 nM) for 6 h and subjected to qRT-PCR. **, *p* < 0.01; ***, *p* < 0.001.

To explore whether posttranslational modifications regulate USP35 nuclear translocation, we found that Ser613 phosphorylation occurred at the highest frequency in USP35 in PhsophoSitePlus (https://www.phosphosite.org). Interestingly, RRRLGS^613^, the motif where Ser613 is located, is conformed to the consensus AKT phosphorylation motif, RXRXXS(T) [32] and is conserved in mammals, except rat and mice (Fig. 6a).

Using antibodies recognizing the phosphorylated AKT consensus peptides, we found that there was robust phosphorylation of USP35^WT^, whereas phosphorylation of USP35^S613A^ was greatly diminished in T-47D cells (Fig. 6b). To test whether Ser613 can be phosphorylated by AKT, Flag-USP35^WT^ or Flag-USP35^S613A^ vector was cotransfected with the activated AKT mutant plasmid into 293 T cells. The result showed that USP35^WT^ was robustly phosphorylated, whereas USP35^S613A^ phosphorylation was lost in the presence of activated AKT (Fig. 6c). Similarly, USP35^S613A^ phosphorylation was reduced compared with USP35^WT^ in MCF-7 cells. Importantly, AKT inhibitor MK2206 inhibited USP35^WT^ phosphorylation in MCF-7 cells (Fig. 6d). Additional experiment demonstrated that AKT coimmunoprecipitated with USP35 (Fig. 6e). These data indicate that AKT interacts with USP35 and phosphorylates USP35 at Ser613.

To explore the effects of Ser613 phosphorylation on USP35 localization and function, MCF-7 and T-47D cells were engineered to express vector alone, Flag-USP35^WT^, Flag-USP35^S613A^, and Flag-USP35^S613E^ (Fig. S5). Immunofluorescent staining showed that USP35^WT^ was localized in the cytoplasm and nucleus, whereas USP35^S613A^ was only present in the cytoplasm in MCF-7 cells. Importantly, the phospho-mimetic mutant USP35^S613E^, was localized in the cytoplasm and nucleus (Fig. 6f, Fig. S6). Furthermore, treatment with AKT inhibitor MK2206 or PI3K inhibitor GDC0941 prevented nuclear translocation of USP35^WT^, respectively (Fig. S7). However, MK2206 did not prevent nuclear localization of USP35^S613E^ (Fig. S11). These results indicate that AKT, a key effector of PI3K, dependent phosphorylation of Ser613 is required and sufficient for nuclear localization of USP35.

To test whether Ser613 phosphorylation affects ERα regulated gene expression, T-47D cells were pretreated with MK2206 before being stimulated with E_2_. qRT-PCR analysis showed that MK2206 blocked USP35-enhanced mRNA levels of the ERα target genes (Fig. 6g). In addition, E_2_-stimulated mRNA levels were enhanced in cells expressing USP35^WT^ or USP35^S613E^ in comparison to cells with vector control. In contrast, expression of USP35^S613A^ failed to enhance E_2_-stimulated expression of these genes in MCF-7 (Fig. 6h) and T-47D (Fig. 6i) cells. Interestingly, MK2206 treatment partially and completely inhibited USP35^S613E^-enhanced ERα target genes mRNA levels in ER+ breast cancer cells (Fig. S12). Furthermore, USP35^WT^ enhanced, whereas USP35^S613A^ failed to enhance, the activity of the C3-luc reporter in 293T17 cells (Fig. S8). Collectively, our data indicate that USP35-S613 phosphorylation by AKT is required, but not sufficient, for USP35 enhancement of ERα target gene expression. AKT may phosphorylate other protein target that acts in concert with USP35 to promote the expression of ERα target genes in ER+ breast cancer cells.

## Discussion

**Fig. 7:**
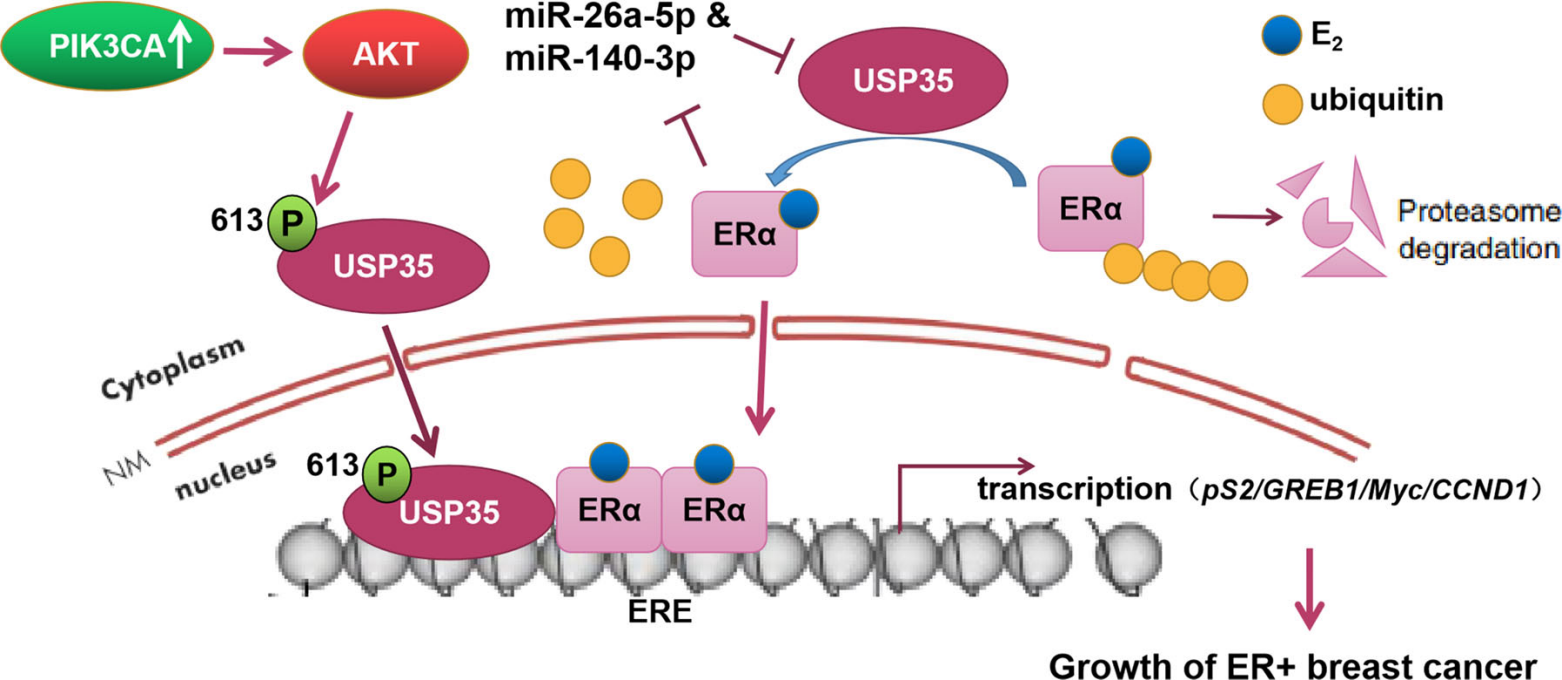
A working model of USP35 action in ER positive breast cancer. USP35 promotes ERα stabilization via deubiquitinating ERα. In addition, AKT phosphorylates USP35 at Ser613, which promotes USP35 nuclear localization, enhancing ERα transcriptional activity. Estrogen increases USP35 expression through inhibiting miR-26a-5p and miR-140-3p expression. This positive feedback loop promotes ER+ breast cancer tumorigenesis.

Our study uncovers a novel mechanism by which USP35 interacts with and promotes the stability and the transcriptional activity of ERα, resulting in the growth of ER+ breast cancer cells. In addition, Akt phosphorylating Ser613 in USP35 is critical for USP35 nuclear translocation and enhancing ERα transcriptional activity (Fig. 7).

Published reports showed that USP35 isoforms elicit different functions in cytosol. While the full length USP35, present in cytosol, promotes mitotic progression by stabilizing Aurora B [24], and cell survival in response to apoptotic stimulus [23], USP35^iso2^ is localized in endoplasmic reticulum, causing endoplasmic reticulum stress and apoptosis [23]. We first noticed nuclear USP35 in breast tumor cells from immunohistochemistry (Fig. 1e). USP35 nuclear staining was positively associated with ER+ breast tumors (data not shown). In addition, immunofluorescent staining results showed that USP35^WT^ or USP35^S613E^, the phosphor-mimetic mutant was present both in the cytosol and the nucleus, whereas the USP35^S613A^ mutant was localized only in the cytosol (Fig. 6f, Fig. S6). Furthermore, AKT inhibitor blocked Ser613 phosphorylation (Fig. 6d), and AKT or PI3K inhibitor prevented nuclear translocation of USP35 (Fig. S7). Functionally, Ser613 phosphorylation was important for USP35 promoting E_2_-stimulated gene expression (Figs. 6h, i) and enhancing the growth of ER+ breast cancer cells (Fig. S9). However, Ser613 phosphorylation did not affect the deubiquitination activity of USP35 against ERα (Fig. S10). Although USP35 increases ERα level, it also seems to function as a transcriptional modulator for genes containing ERE. Supporting this notion, we found that USP35 was present basally in the DNA regions containing ERE in *pS2* and *GREB1*. E_2_ stimulation further enhanced USP35 recruitment (Fig. 5d) and concomitant recruitment of USP35 and ERα (Fig. 5g) to these regions. USP35 overexpression promoted the basal and E_2_-stimulated recruitment of ERα (Fig. 5e), whereas knockdown of USP35 (Fig. 5f) only inhibited the E_2_-stimulated recruitment of ERα to these ERE-containing regions. Interestingly, other deubiquitinases activate transcription by deubiquitinating Histone 2A [33]. Future studies are required to elucidate how USP35 functions as a putative transcriptional cofactor.

Our study clearly demonstrated that USP35 promotes the growth of ER+ breast cancer cells in vitro and in vivo, which is consistent with higher USP35 expression in breast tumors compared with adjacent normal breast tissues (Figs. 1a, d). In addition, breast cancer with high USP35 expression or gene amplification is associated with poor survival (Figs. 1f, g, h). USP35 promotes the growth of ER+ breast cancer cells by regulating the G1 to S phase transition (Fig. 3d), which is in agreement with USP35 being a cell-cycle regulator in mitosis [24]. However, our result is in contrast with a previous report, which showed that USP35 inhibited the growth of lung cancer cells by stabilizing the ABIN-2 [25]. It remains to be determined whether this tumor-suppressor function of USP35 is the action of USP35^iso2^ or the cancer type-specific action of USP35.

Ubiquitination affects the stability and function of ERα in breast cancer cells. Our study revealed that USP35 promotes ERα protein stability by interacting and deubiquitinating ERα. Using different deletion mutant of USP35, we found that USP35 interacts with ERα via its USP35 domain (data not shown). The catalytic cysteine450 is required for ERα deubiquitination (Fig. 4h, Fig. S4b). Polyubiquitination of ERα usually leads to ERα degradation via the proteasomal pathway [34]. For example, ERα was ubiquitinated and degraded by Ddb1-cullin4-associated-factor1 [35]. In addition, tumor suppressor BRCA1 in a complex with BARD1 can also ubiquitinate and degrade ERα [36]. However, monoubiquination of ERα by various E3 ubiquitin ligases has been shown to increase ERα stability [17, 37, 38]. Therefore, USP35 likely deubiquitinates degradation-associated ubiquination events to promote ERα stability. A recent report showed that USP22, a constitutive nuclear deubiquitinase, can promote ERα stability by deubiquitinating ERα in the nucleus of breast cancer cells [39].

Although *USP35* is amplified in ~9% of the breast cancer (Fig. 1g), our study revealed that gene amplification is not the major mechanism for higher USP35 expression in breast cancer, since USP35 mRNA levels are significantly higher in luminal (ER+) breast cancer (Fig. 1b), which account for ~70% of the breast cancer, in comparison to TNBC and HER2+ breast cancer. There is a subgroup of ER+ with high level of USP35 mRNA (Fig. 1b), which maybe the result of *USP35* amplification, since *USP35* was amplified in ~13% of ER+ breast cancer (Fig. 1h). Importantly, our data showed that estrogen increases USP35 protein expression by downregulating *miR-140-3p* and *miR-26a-5p* levels in ER+ breast cancer cells (Fig. 2). Estrogen inhibition of *miR-140-3p* and *miR-26a-5p* expression in ER+ breast cancer cells were reported previously [28, 30]. Our study demonstrated that *USP35* is the bona fide target for *miR-140-3p* and *miR-26-5p* in ER+ breast cancer cells (Fig. 2d). Interestingly, analysis of data in TCGA revealed that *USP35* mRNA and *miR-140-3p* levels were inversely correlated in breast cancer (data not shown). Since USP35 promotes ERα stability, our data indicate that USP35 forms a positive feedback loop with ERα, promoting tumorigenesis of ER+ breast cancer.

*PIK3CA*-activating mutations occur in ~40% of patients with hormone receptor-positive breast cancer. Our results strongly suggest that *PIK3CA* mutants can signal to USP35 through AKT phosphorylating Ser613 to promote the growth of ER+ breast cancer cells. Consistent with this notion, a recent study indicated that ER+ breast cancer with *PIK3CA* mutations and reduced survival is associated with amplification at the *11q13-14* locus [12] that harbors *USP35*. Interestingly, two of the ER+ breast cancer cell lines used in our study, MCF-7 and T-47D, contain hotspot mutations in PI3K, E542K, and H1047R respectively [40]. Thus, ER+ breast cancer with *PIK3CA* mutations and higher USP35 expression may have poor survival. Treatment with PI3Kα specific inhibitor alpelisib together fulvestrant increased progression-free survival of ER+ advanced breast cancer with *PIK3CA* mutations that had received endocrine therapy previously [10]. Consistent with this report, our data showed that knockdown of USP35 increased the inhibitory response to tamoxifen and fulvestrant treatment in MCF7 cells that harbor PI3Kα-E542K mutation (Fig. 3g), and in T-47D cells that have PI3Kα-H1047R mutation (Fig. 3h). A recent study indicated that treatment with the PI3Kα inhibitor induced an adaptive enhanced ERα signaling by activating KMT2D, a H3K4 methyltransferase in ER+ breast cancer cells with *PIK3CA* mutation [41], indicating therapeutic resistance to PI3Kα inhibitor. Therefore, a putative small molecule inhibitor for USP35 may help overcome resistance to therapy with PI3K α inhibitor for ER+ breast cancer with *PIK3CA* mutations or hyper-activation of the PI3K pathway in the future.

In summary, our study reveals that higher USP35 expression is associated with ER+ breast cancer and poor prognosis. USP35 promotes tumorigenesis of ER+ breast cancer by enhancing the stability and transcriptional activity of ERα, and increases resistance of ER+ breast cancer cells to endocrine therapy. AKT phosphorylation of USP35 is critical for USP35′s action in ER+ breast cancer cells. USP35 should be a potential therapeutic target for ER+ breast cancer that develops resistance to standard targeted therapies.

## Materials and methods

### Cell lines and reagents

MCF-7, T-47D, ZR-75-1, HCC1954, BT-474, Hs578T, and MDA-MB-231 human breast cancer cell lines and 293T17 were obtained from the American Type Culture Collection (ATCC, Maryland, USA). Human breast epithelial cell line MCF10A was a gift from Dr. Joan Brugge, Harvard Medical School. MCF-7, Hs578T, and 293T17 cells were cultured in DMEM medium (Gibco, California, USA) with 5% fetal bovine serum (FBS) (Lonsera, Australia), while T-47D and ZR-75-1 cells were cultured in DMEM with 10% FBS, supplemented with 1% penicillin/streptomycin (Beyotime Biotechnology, Jiangsu, China). HCC1954 cells were cultured in RPMI1640 medium (Gibco) with 10% FBS, 1% penicillin/streptomycin. BT474 cells were cultured in D/F12 medium (Gibco) with 10% FBS and 1% penicillin/streptomycin. MDA-MB-231 were cultured in MEM medium (Gibco) with 5% FBS, 1% penicillin/streptomycin, and 1.8 μg/mL insulin (Solarbio, Beijing, China). MCF10A was cultured in DMEM/F12 medium with 5% horse serum (Hyclone, Utah, USA), 1% penicillin/streptomycin, 20 ng/mL EGF (Peprotech, New Jersey, USA), 0.5 μg/mL hydrocortisone (Solarbio), and 10 μg/mL insulin. DMEM medium without phenol red, dextran-coated charcoal, and 17β-estradiol were purchased from sigma (St. Louis, USA) and dissolved in ethanol. Tamoxifen and fulvestrant were purchased from Selleck (Texas, USA). For hormone starvation of ER+ breast cancer cells, cells were cultured in 5% fetal calf serum pretreated with dextran-coated charcoal and phenol red-free DMEM for 3 days or the indicated time. MG132, MK2206, and GDC0941 were purchased from Selleck. Cycloheximide was purchased from Sigma (Darmstadt, Germany).

### Plasmids

pcDNA3.1(+)−3xFlag-USP35 plasmid was a gift from Dr. Peter K Kim, the Hospital for Sick Children, Toronto [22]. To construct pBabe-puro-Flag-USP35 plasmid, pcDNA3.1-3xFlag-USP35 DNA was digested with *Xho*I and *Bam*HI and ligated to *Sal*I and *Bam*HI linearized pBabe-puro plasmid. pcDNA3.1(+)-Flag-USP35-C450A was generated from pcDNA3.1-Flag-USP35 plasmid. pBabe-puro-Flag-USP35-S613A and S613E plasmids were generated from pBabe-puro-Flag-USP35 as a template using site-directed mutagenesis PCR. USP35-3′UTR was amplified from MCF-7 cDNA by PCR and subcloned into the pmirGLO vector downstream of the firefly luciferase cDNA. pmirGLO-USP35-3′UTR with the predicted miR-26a-5p and miR-140-3p binding site mutations were generated using site-directed mutagenesis PCR. USP35-shRNA1 and USP35-shRNA2 (sequences from sigma) were subcloned into the *Eco*RI and *Age*I digested pLKO.1 vector (a gift from Dr. Hezhi Fang, Wenzhou Medical University). All primer sequences for PCR amplification and cloning in this study were listed in the Supplementary information. All of the generated plasmids were verified by DNA sequencing (GENEWIZ, Jiangsu, China). The estrogen responsive C3-luciferase reporter plasmid was purchased from addgene (MA, USA). ERα-Tag2 and HA-ubiquitin expression plasmids were gifts from Dr. Binhua P. Zhou, University of Kentucky. Myr-AKT1-ER plasmid was a gift from Dr. Lewis Cantley (Harvard Medical School).

### Retrovirus/lentivirus production and viral infection

The production of retroviruses and lentiviruses and viral infection of cells were performed essentially as described [42, 43].

### Transient transfection of cells with miRNA mimics

Scramble non-specific control (NC) miRNA, miR-26a-5p, and miR-140-3p mimics were purchased from GenePharma (Shanghai, China). Breast cancer cells plated in 24-well plate were transfected with 60 nM miRNA mimic using Lipofectamine 2000 reagent (Invitrogen) according to the manufacturer’s instructions. Forty-eight to 72 h after transfection, cells were harvested and subjected to various assays.

### qRT-PCR

Total RNAs were isolated from cells using the TRIZOL reagent (Invitrogen) according to the manufacturer’s instructions. cDNA was synthesized from 1 μg of purified RNA using HiScript II Q RT SuperMix (Vazyme, Jiangsu, China) and subjected to quantitative real-time PCR (qRT-PCR) for analyzing relative mRNA level of specific genes with proper primers using ChamQ Universal SYBR qPCR Master Mix (Vazyme).

### Immunohistochemistry

Formalin-fixed and paraffin-embedded (FFPE) primary breast tumor samples were from the Pathology Department at The First Affiliated Hospital of Wenzhou Medical University. FFPE breast tumor sections (4 μm thickness) were subjected to USP35 immunohistochemistry (IHC) as essentially described [42] with some modifications. Antigen retrieval was performed in 10 mM citrate buffer, pH 6.0 using pressure cooker (at 125 °C for 5 min). Rabbit anti-USP35 polyclonal antibodies (ab128592, abcam, Cambridge, UK) were used at 1:500 dilution. USP35 IHC staining was scored by two pathologists blindly in the following way. The staining intensity was scored in four levels (0, 1, 2, and 3). The percentage of positive staining cells was scored in 6 levels (0% = 0, <1% = 1, 1–10% = 2, 11–33% = 3, 34–66% = 4, 67–100% = 5). USP35 IHC score was calculated as the sum of the score for staining intensity and the score for percentage of positive stained cells blindly by two individuals.

### Cell colony formation assay

Breast cancer cells were cultured in 24-well tissue culture plates for a week to form colonies before being fixed with 10% neutral formalin, stained with 0.5% crystal violet solution, and having the dye extracted by adding 10% acetic acid. The absorbance at 540 nm was measured using a Varioskan flash microplate reader.

### Soft agar colony assay

Breast cancer cells resuspended in medium containing 0.3% agar were added to the 6-well plates prefilled with solidified 0.5% agar, incubated in tissue culture incubator overnight, and fed with 0.5 mL fresh medium. The plates were replenished with fresh medium every 3 days until the colonies became larger than 50 μm in diameter. Images of the colonies in five random 4 × fields were captured by the Nikon Eclipse TI-S microscope. Colonies with a diameter greater than 50 μm were quantified using the NIS-Element software. The average number of colonies in five random fields was determined for each well.

### Cell cycle analysis

MCF-7 cells with Con-shRNA, USP35-shRNA1, and USP35-shRNA2 were seeded in 6-well plates for 3 days before being harvested, fixed in 70% ethanol overnight, and stained by propidium iodide according to the manufacturer’s instructions (Beyotime). The BD LSR II flow cytometer (BD Bioscience, Franklin Lakes, NJ, USA) was used to analyze the DNA contents in different cell cycle population.

### MCF-7 orthotopic xenograft model

MCF-7 cells (5 × 10^6^) with vector and USP35 overexpression were suspended in a 200 μL mixture of medium and matrigel (BD Biosciences) (1:1) and injected into both sides of the fourth mammary fat pad of 5-week-old female NCG mice (GemPharmatech, Jiangsu, China) (5 mice each group). Mice were implanted with the estrogen pellets (60-day release, 1.5 mg/pellet, Innovative Research of America) on the back (near the neck) 2 days before cell injection. MCF-7 tumor growth in NCG mice was monitored by measuring the tumor size with a Vernier caliper once a week. Mice were sacrificed 7 weeks after cell injection. Tumor volume was calculated using the formula V = (A × B^2^)/2, in which A is the long diameter (mm) and B is the short diameter (mm). Tumor tissues were fixed in 10% formalin solution for 24 h, embedded in paraffin, and subjected to immunohistochemistry.

### Immunoprecipitation and immunoblotting

Cells were washed twice in cold PBS and lysed in IP buffer (50 mM Tris, pH 7.4, 150 mM NaCl, 1% Triton X-100, 20 mM beta-glycerol) plus 10 mM NaF, 2 mM Na_3_VO_4_, and protease inhibitor cocktail (Bimake, Houston, USA). Whole-cell lysates were clarified by centrifugation and incubated with the appropriate primary antibodies or M2-Flag beads (Sigma) overnight at 4 °C. Antibody-bound proteins were precipitated with protein A agarose (REPLIGEN, Boston, USA). Protein A agarose or M2-beads were washed five times with lysis buffer and then eluted in 1 × SDS sample loading buffer. Eluted proteins were separated by SDS PAGE, transferred to PVDF membranes (Millipore), immunoblotted with the appropriate primary antibodies, and horseradish peroxidase-conjugated secondary antibodies (Jackson Immunoresearch, USA), and detected by Enhanced Chemiluminescence. USP35 rabbit antibody (ab128592, 1:2000) was from Abcam. USP35 rabbit polyclonal antibodies for immunoprecipitation were generated against the His-tagged USP35 C-terminus (aa 856–1018) by HUABIO (Hangzhou, Zhejiang, China) and affinity purified using the His-tagged USP35 C-terminus as the affinity reagent. Mouse antibody against ERα (sc8005) was from Santa Cruz Biotechnology (Dallas, Texas, USA). Rabbit antibodies against ERα (8644, 1:1000), pAKT substrate (RXRXXS/T) (10001, 1:1000), AKT (4691, 1:1000) and mouse antibodies against α-tubulin (3873, 1:5000), β-actin (3700, 1:5000) were from Cell Signaling Technology. Rabbit antibody against HA tag (AF0039, 1:1000) and mouse antibody against Flag tag (AF519, 1:1000) were from Beyotime Biotechnology.

### Deubiquitination assay

HA-ERα and HA-ubiquitin plasmids were cotransfected with vector or Flag-USP35 plasmid into 293T17 cells. Forty-eight hours later, cells were treated with MG132 (10 μM) for 6 hours before being harvested and lysed with IP buffer. Clarified cell lysates were incubated with anti-ERα antibody (sc8005) at 4 °C overnight and with added protein A agarose for one additional hour. The protein A agaroses were washed five times with lysis buffer and eluted with 1 × SDS sample loading buffer, then subjected to immunoblotting analysis.

### Immunofluorescence staining

Cells were grown on the coverslip, fixed with 4% paraformaldehyde, permeabilized with 0.2% Triton X-100, washed, blocked with 10% normal donkey serum, and incubated with anti-Flag antibody (1:500, AF519, Beyotime Biotechnology). TRITC-anti-mouse secondary antibodies (1:40) (Jackson Immunoresearch, West Grove, PA) and Hoechst dye (5 μg/mL) (Invitrogen) were then added to the cells. Images of stained cells were captured using the Nikon A1 confocal microscope.

### Chromatin immunoprecipitation (ChIP) assay and reChIP assay

ChIP was performed using the Chromatin Immunoprecipitation (ChIP) Assay Kit (Upstate cell signaling solutions, Catalog #17–295). ReChIP assay was performed as described [44] with some modifications. Briefly, cells were starved in medium containing 5% charcoal-stripped serum for 3 days and treated with E_2_ (10 nM) for 0, 15 and 30 minutes. Cells (3 × 10^6^) were then cross-linked using 1% formaldehyde in condition cell medium for 10 minutes at RT, then were subsequently washed twice with PBS. Nuclei preparation and chromatin digestion were performed according to the manufacturer’s instructions. Digested chromatin DNAs were incubated with USP35 antibodies (6 μg/sample), normal rabbit IgG (Invitrogen 02–6102, 6 μg/sample), or ERα antibody (sc-8005, 1.2 μg/sample). ChIP DNA was eluted, purified, and analyzed by real time PCR using primers detecting pS2 and GREB1 enhancer regions containing estrogen-responsive element. Relative occupancy values were determined by calculating the ratios of the amount of immunoprecipitated DNA to that of the input sample. For ChIP with anti-USP35 and anti-ERα, the values were normalized to the values of control IgG, defined as 1. For reChIP assay, digested chromatin DNAs were incubated first with USP35 antibodies and protein A agarose bead, eluted with buffer containing 10 mM Tris-HCl, 2 mM EDTA, 2% SDS, 15 mM DTT and protease inhibitors, diluted 20-fold with immunoprecipitation buffer, and were incubated with ERα antibody, then followed by qPCR.

### Luciferase assay

MCF-7 and ZR-75-1 cells with vector alone or USP35 overexpression were cotransfected with C3-luciferase (firefly) reporter and TK-renilla luciferase plasmids (C3-luciferase plasmid: TK-renilla plasmid = 20:1). 293T17 cells were cotransfected with the C3-luciferase reporter/TK-renilla plasmids together with vector alone or pcDNA3.1-USP35-WT and/or ERα expression vector. Transfected cells were starved in 5% charcoal stripped serum for 2 days, stimulated with 10 nM E_2_ for 24 h before being subjected to luciferase activity assay. MCF-7 cells were cotransfected with USP35 3′UTR-WT luciferase or USP35 3′UTR-mutant luciferase reporter plasmid together with scramble NC, miR-26a-5p and/or miR-140-3p mimics for 3 days before being subjected to luciferase activity assay using the Luciferase Assay System Kit (Promega, Wisconsin, USA) according to the manufacturer’s instructions. All assays were performed in triplicate, and the luciferase activities were normalized against TK-renilla activities.

### Statistics and reproducibility

Statistical significance was calculated by using GraphPad Prism 7 in this study. The log-rank Mantel−Cox test was used to analyze the effects of *USP35* on overall survival for breast cancer. One-way analysis of variance (ANOVA) was used to compare differences among multiple sample groups. Unpaired two-tailed Student’s *t*-test was used to compare data between two groups, except in the case of analyzing tumor and adjacent normal tissues where the paired Student’s *t*-test was used. *p* < 0.05 was considered statistically significant. All experiments were repeated at least three times.

## Supplementary information

supplement figure

supplementary information

checklist
