# Supplementary material for: USP35, regulated by estrogen and AKT, promotes breast tumorigenesis by stabilizing and enhancing transcriptional activity of estrogen receptor α
Source: Cell Death Dis. 2021 Jun 15;12(6):619. doi: 10.1038/s41419-021-03904-4 (PMC8206120; doi:10.1038/s41419-021-03904-4)

**Supplementary Figures**

**Figure. S1**

**
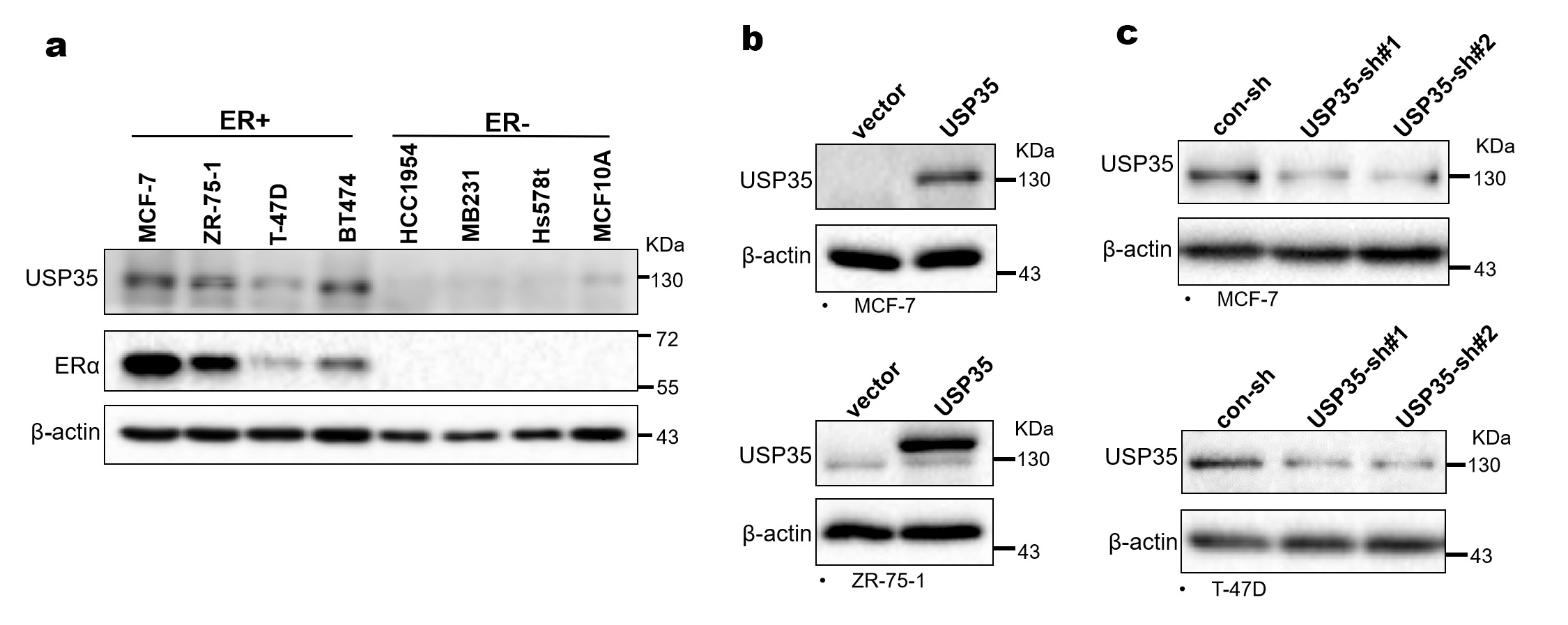
**

**Figure. S2**


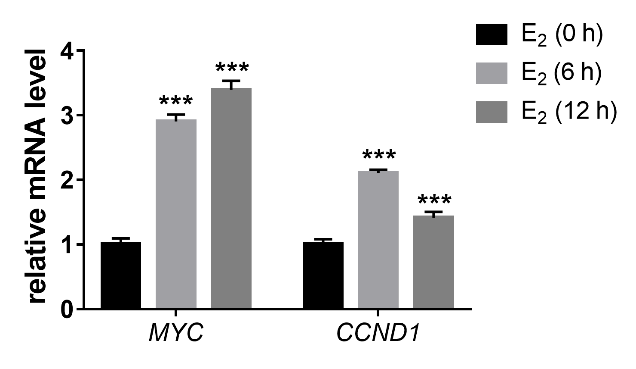


**Figure. S3**

**
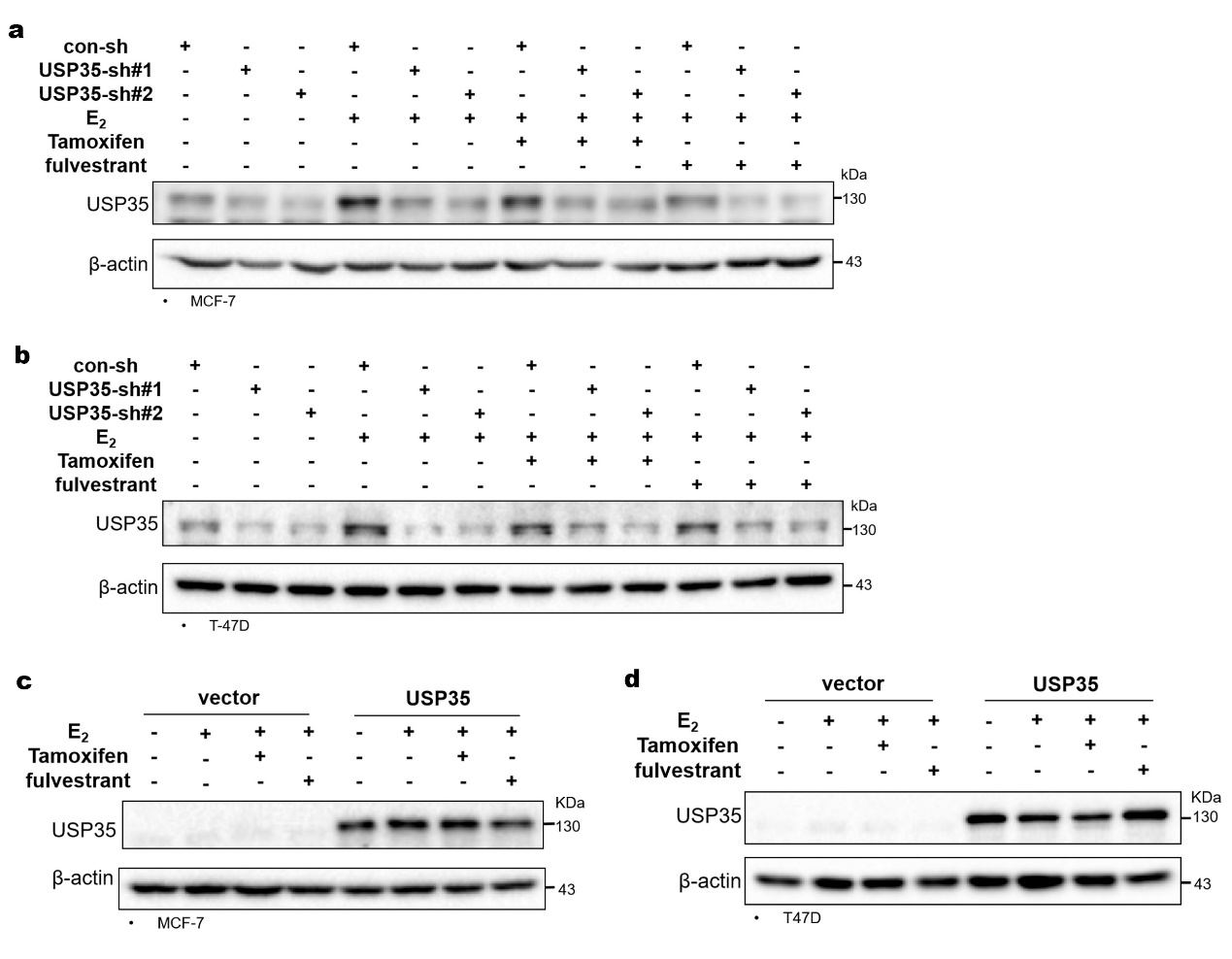
**

**Figure. S4**


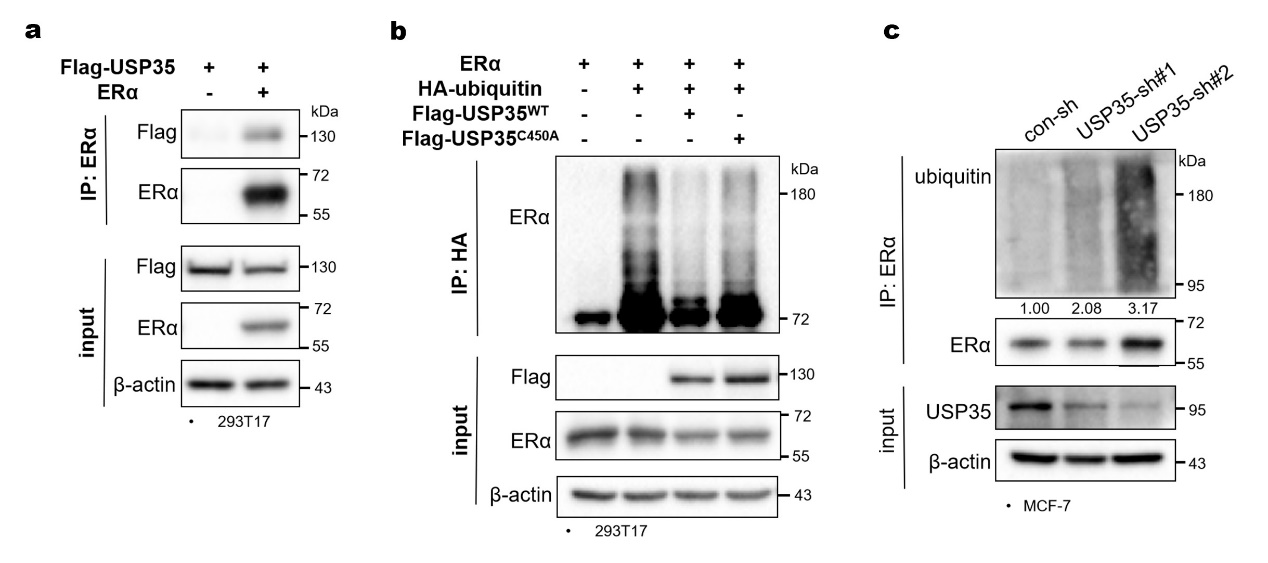


**Figure. S5**


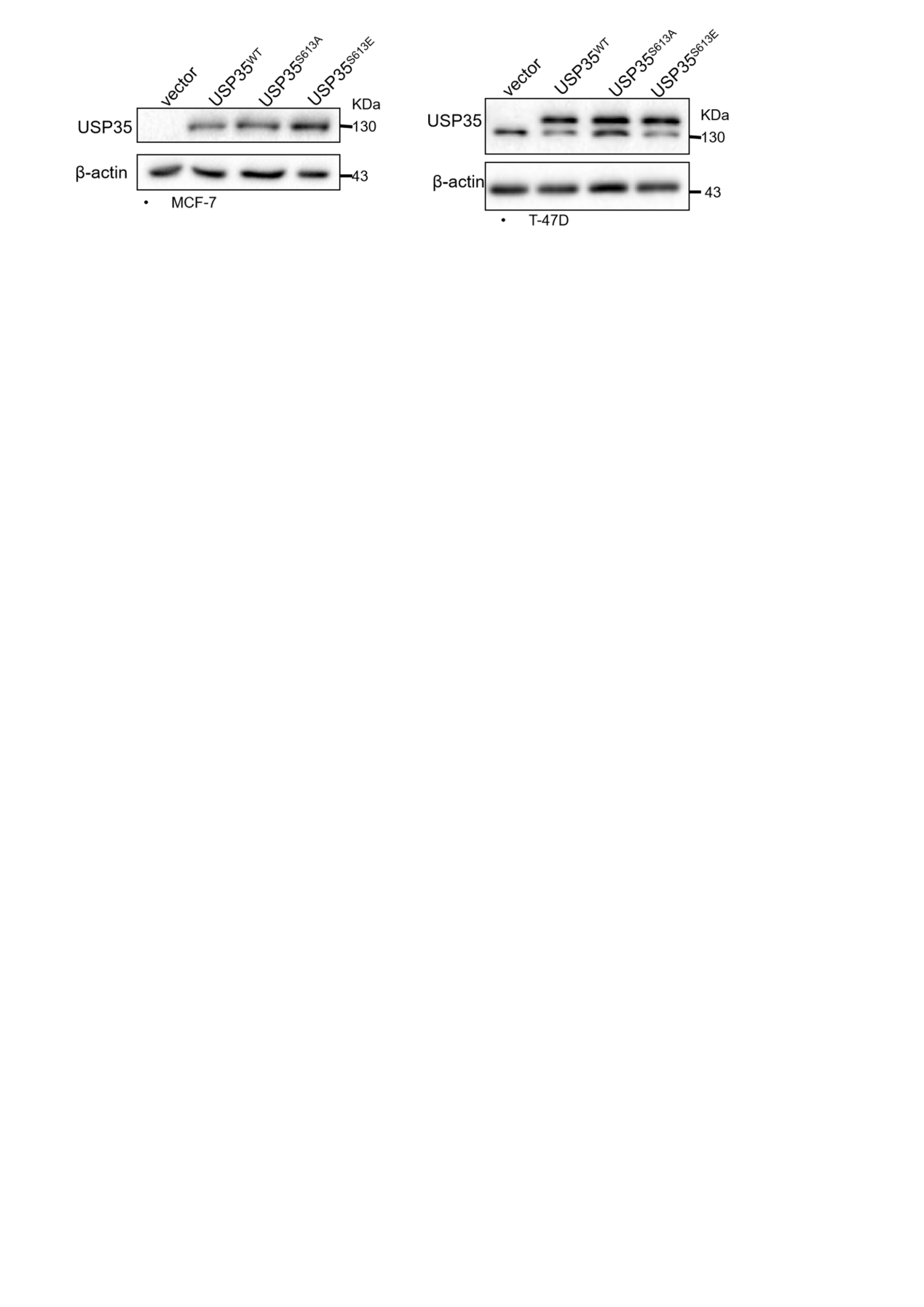


**Figure. S6**


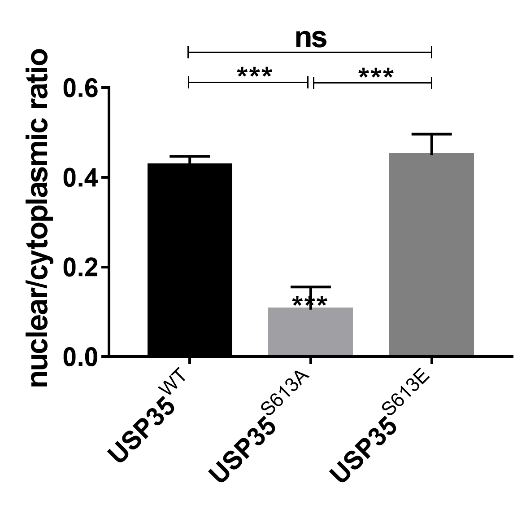


**Figure. S7**


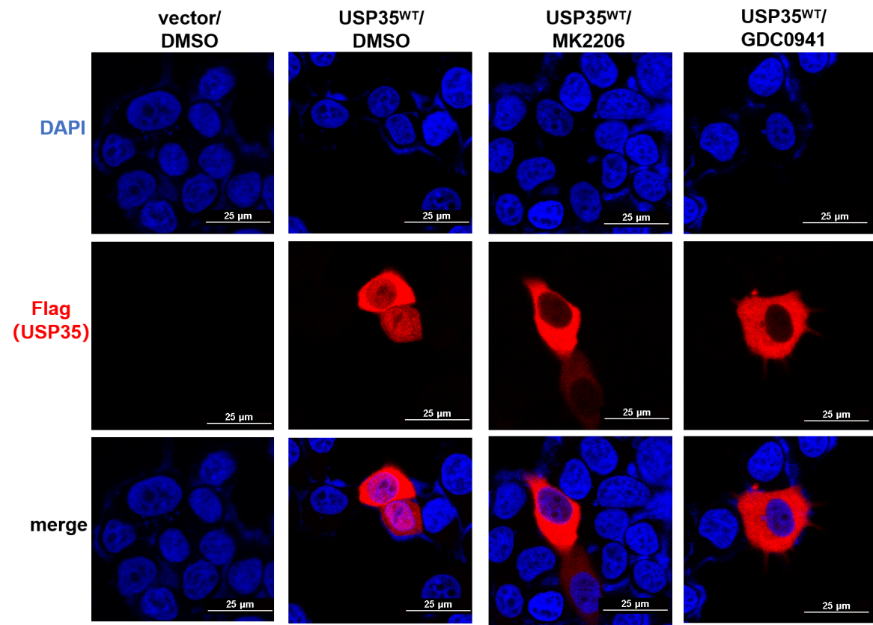


**Figure. S8**

**a**


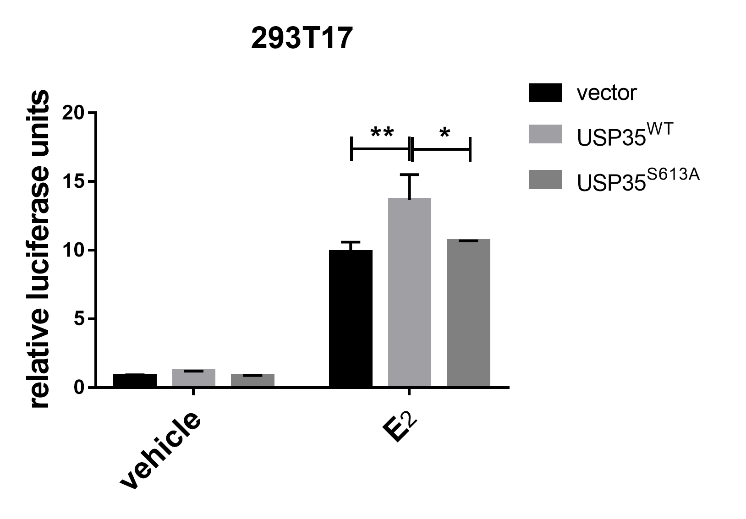


**b**


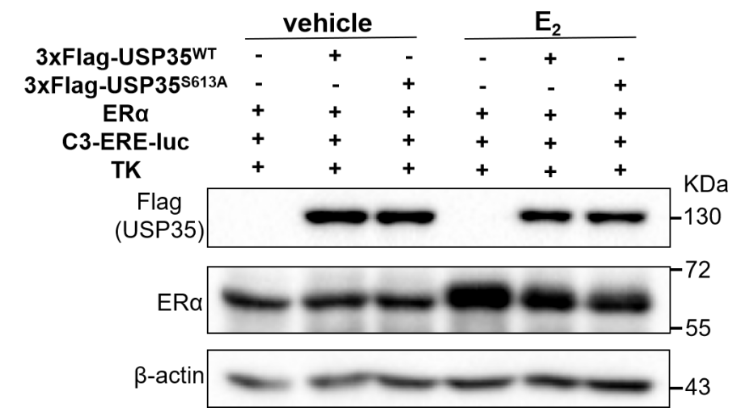


**Figure. S9**


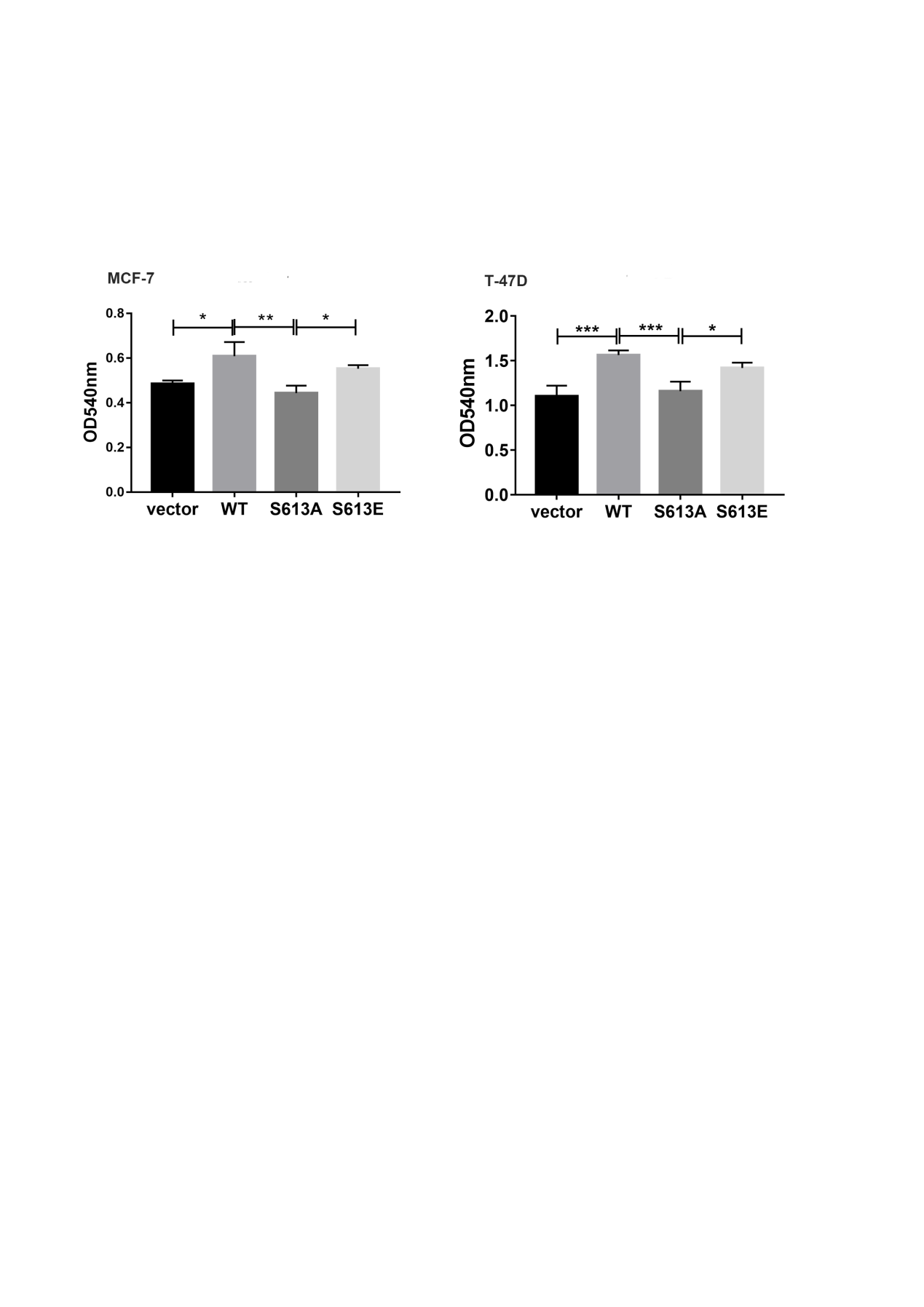


**Figure. S10**


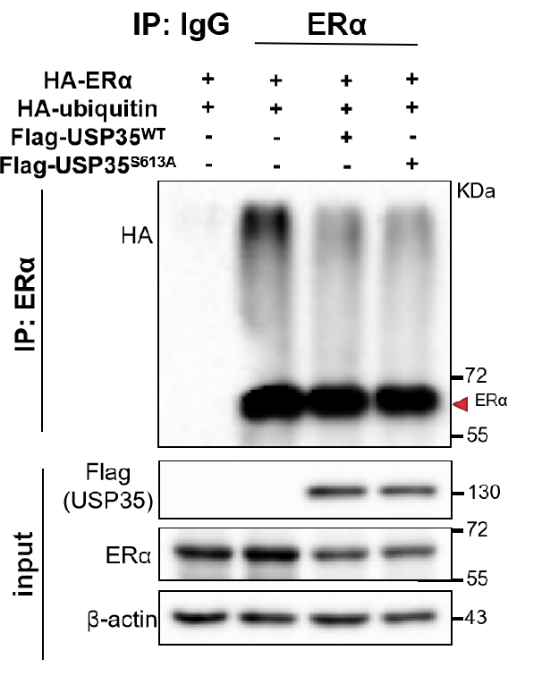


**Figure. S11**


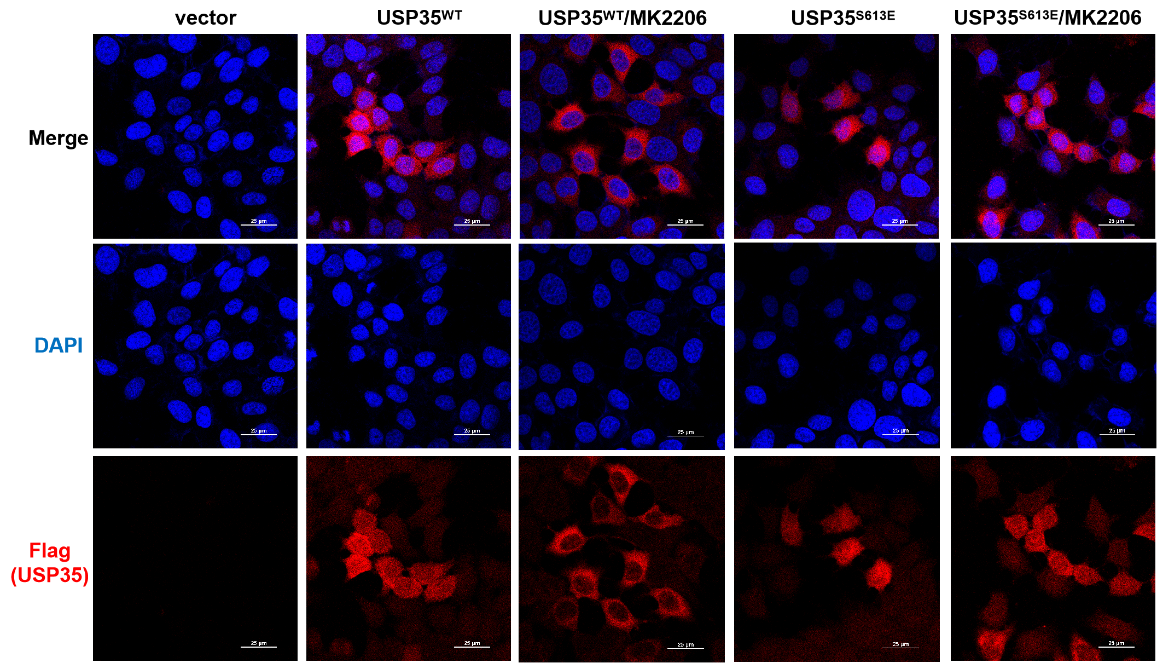


**Figure. S12**


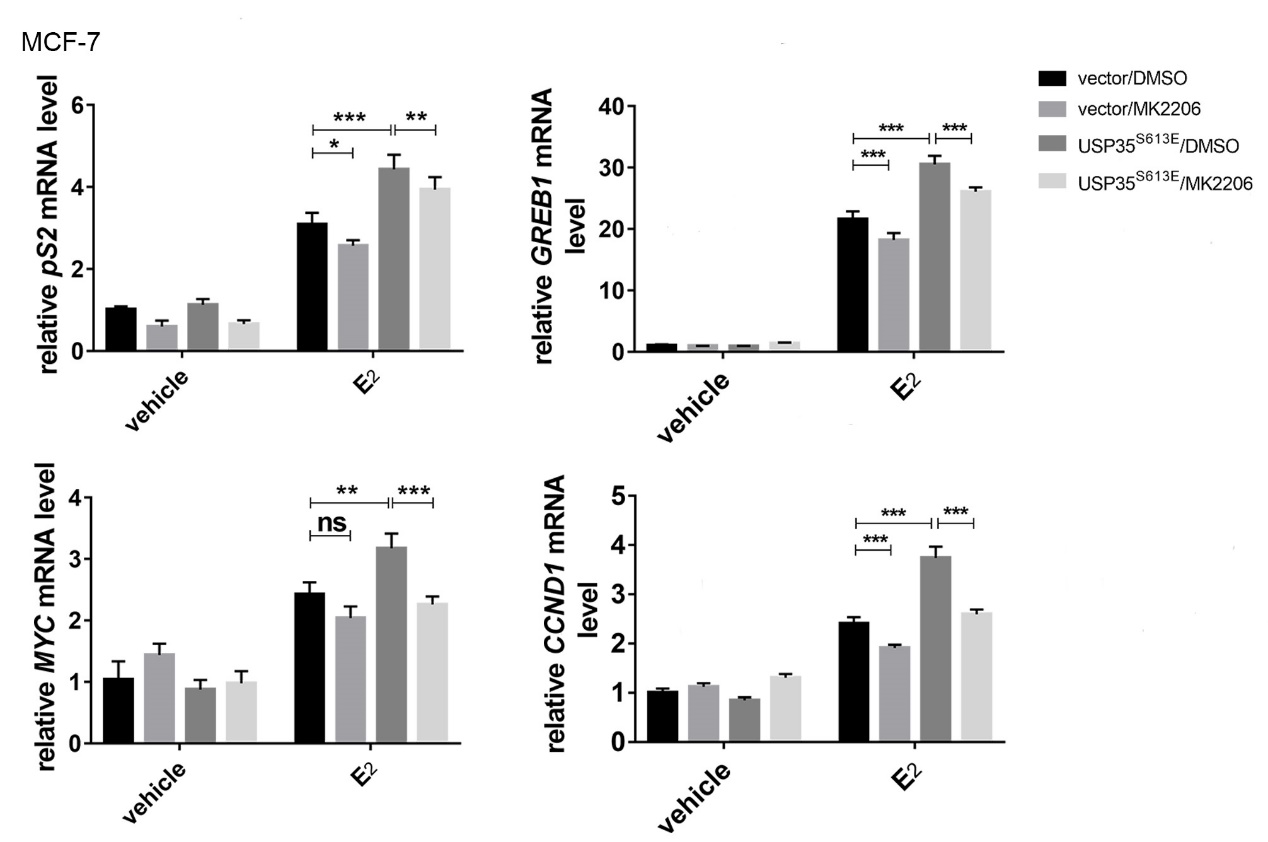

Supplement: Supplementary file 1 — supplement figure [file 41419_2021_3904_MOESM1_ESM.docx]
