# Supplementary material for: USP35, regulated by estrogen and AKT, promotes breast tumorigenesis by stabilizing and enhancing transcriptional activity of estrogen receptor α
Source: Cell Death Dis. 2021 Jun 15;12(6):619. doi: 10.1038/s41419-021-03904-4 (PMC8206120; doi:10.1038/s41419-021-03904-4)
Supplement: Supplementary file 2 — supplementary information [file 41419_2021_3904_MOESM2_ESM.doc]

**Supplementary Information**

**Sequence information for all the primers used in the study**

**Two different *USP35* shRNAs:**

USP35-sh#1: 5＇-AGTCAGACACGGGCAAGATTG-3＇

USP35-sh#2: 5＇-CCGACTGCTGTACGGTATAAA-3＇

**The following primer pairs were used for PCR cloning of *USP35-*3’UTR:**

Forward: 5＇-ATAGAGCTCTGTGAACCTGCTGCCAACCTGACC-3＇

Reverse: 5＇-GGCGTCTAGATTGCTTAGGCTTACTTTTATTTATTTATG-3＇

**The following primer pairs were used for site-directed mutagenesis PCR:**

**USP35-C450A**

Forward: 5＇-CACAGCCTATGTCAACAGCATCCTTCAG-3＇

Reverse: 5＇-CATAGGCTGTGTTGCCCAGGTTGATGAG-3＇

**USP35-S613A**

Forward: 5＇-CTGGGCGCTGTGATGCGCCCCACAGAAGAC-3＇

Reverse: 5＇-CATCACAGCGCCCAGGCGGCGGCGGCGACAG-3＇

**USP35-S613E**

Forward: 5＇-CTGGGCGAAGTGATGCGCCCCACAGAAGACATCACAG-3＇

Reverse: 5＇-CATCACTTCGCCCAGGCGGCGGCGGCGACAG-3＇

***USP35-*3’UTR miR-26a binding site mutations**

(228-1) Forward: 5＇-GTCCTAGTTAGCCTGTAGCAGCCGAGATGGGC-3＇

Reverse: 5＇-CTGCTACAGGCTAACTAGGACACTTGGGTGTAAAG-3＇

(228-2) Forward: 5＇-CAAGTGCCTTAGTTAGACTATAGCAGCCGAGATG-3＇

Reverse: 5＇-CTGCTATAGTCTAACTAAGGCACTTGGGTGTAAAG-3＇

***USP35-* 3’UTR miR-140-3p binding site mutation**

1. Forward: 5＇-GGGAAGTTATGCAGGCAGGCCCTACCAAGAGG-3＇

Reverse: 5＇-CTGCCTGCATAACTTCCCTCAGGCCCTACCTG-3＇

1. Forward: 5＇-GGAAGCTTATGCGCTCCTCTGAGCAGTTGGCC-3＇

Reverse: 5＇-GGAGCGCATAAGCTTCCTAAGAGCCCACACTTC-3＇

**The following primer pairs were used for detecting mRNA levels by quantitative real-time PCR:**

***pS2***

Forward: 5＇-CATCGACGTCCCTCCAGAAGAG-3＇

Reverse: 5＇-CTCTGGGACTAATCACCGTGCTG-3＇

***GREB1***

Forward: 5＇-TTTTCAACGGCAAAGATTCC-3＇

Reverse: 5＇-ATGCCTGCGCTCTCATACTT-3＇

***Myc***

Forward: 5＇-ATGCCCCTCAACGTTAGC-3＇

Reverse: 5＇-AGCTCGCTCTGCTGCTGC-3＇

***CCND1***

Forward: 5＇-GCGAGGAACAGAAGTGC-3＇

Reverse: 5＇-GAGTTGTCGGTGTAGATGC-3＇

***GAPDH***

Forward: 5＇-CAGGAGGCATTGCTGATGAT-3＇

Reverse: 5＇-GAAGGCTGGGGCTCATTT-3＇

**Primer pairs used for quantitative real-time PCR for *pS2* and *GREB1* after ChIP were as described(1, 2):**

***pS2*(1)**

Forward: 5＇-CCCGTGAGCCACTGTTGTC-3＇

Reverse: 5＇-CCTCCCGCCAGGGTAAATAC-3＇

***GREB1*(2)**

Forward: 5＇-GAAGGGCAGAGCTGATAACG-3＇

Reverse: 5＇-GACCCAGTTGCCACACTTTT-3＇

References

1. Curtis CD, Likhite VS, McLeod IX, Yates JR, Nardulli AM. Interaction of the tumor metastasis suppressor nonmetastatic protein 23 homologue H1 and estrogen receptor alpha alters estrogen-responsive gene expression. Cancer Res. 2007;67(21):10600-7.

2. Hurtado A, Holmes KA, Ross-Innes CS, Schmidt D, Carroll JS. FOXA1 is a key determinant of estrogen receptor function and endocrine response. Nat Genet. 2011;43(1):27-33.

**Supplementary figure legends**

Figure S1 Expression of USP35 in breast cancer cell lines. (a) USP35 protein level was higher in ER+ than in ER- breast cancer cell lines. (b, c) USP35 levels in MCF-7 and ZR-75-1 cells stably expressing vector or USP35 (b), and in MCF-7 and T-47D cells infected with lentiviruses expressing con-sh and two different USP35 shRNAs (sh#1 and sh#2) (c). con-sh: scramble shRNA control.

Figure S2. Estrogen treatment increases mRNA levels of ERα targeted genes. MCF-7 cells were starved and treated with 10 nM E_2_ for indicated times, and subjected to qPCR analysis for *MYC* and *CCND1* mRNA. ***, *p*<0.001.

Figure S3. USP35 protein levels in the indicated ER+ breast cancer cell lines. MCF-7 cells (**a**) and T-47D cells (**b**) expressing control-shRNA and USP35-shRNAs (sh#1, sh#2) were hormone-starved for 3 d and then treated with 10 nM E_2_, together without and with 5 μM Tamoxifen or 1 μM fulvestrant for 3 d before being subjected to western blot. MCF-7 (**c**) and T-47D (**d**) cells with vector or USP35 overexpression were treated and analyzed as described above.

Figure S4. USP35 interacts with and deubiquitinates ERα. (**a**) USP35 interacts with ERα. 293T17 cells were co-transfected with 3xFlag-USP35 and ERα plasmids. Cell lysates were immunoprecipitated with anti-ERα antibody followed by immunoblotting with the indicated antibodies. (**b**) USP35 promotes ERα deubiquitination. 293T17 cells were cotransfected with HA-ubiquitin and ERα plasmids together with 3×Flag-USP35^WT^ or 3×Flag-USP35^C450A^ plasmid. Cells were treated with MG132 (10 μM) for 6 h before being subjected to immunoprecipitation using the anti-HA antibody. (**c**) USP35 knockdown increases ERα ubiquitination. MCF-7 cells expressing control-shRNA and USP35-shRNAs (sh#1, sh#2) were treated with MG132 (10 μM) for 6 h before being subjected to immunoprecipitation with anti-ERα antibody followed by immunoblotting with the indicated antibodies.

Figure S5. Expression of exogenous USP35 wild type and mutants in MCF-7 and T-47D cells. Cells stably expressing vector or Flag-USP35^WT^, Flag-USP35^S613A^, Flag-USP35^S613E^ were immunoblotted with USP35 and β-actin antibodies.

Figure S6. Ser613 is critical for nuclear translocation of USP35 in breast cancer cells. MCF-7 cells expressing Flag-USP35^WT^, Flag-USP35^S613A^ and Flag-USP35^S613E^ were immunostained with anti-Flag antibody and examined by confocal microscopy. Fluorescent intensity in the nucleus and cytoplasm was analyzed by Image J. Ten cells for each cell line were analyzed and the ratio of nuclear and cytoplasmic intensity was calculated and shown. ***, *p*<0.001.

Figure S7. Inhibitors of AKT and PI3K block nuclear translocation of USP35 in breast cancer cells. MCF-7 cells expressing vector, and Flag-tagged USP35^WT^ were treated with DMSO, 500 nM MK2206 (AKT inhibitor), and 1 μΜ GDC0941 (PI3K inhibitor) for 6 h before being immunostained with anti-Flag antibody (red) and DAPI (blue). Immunofluorescent images were captured by confocal microscopy. Bar=25 μm.

Figure S8. USP35 Ser613 is important for USP35 enhancement of E_2_-induced ERα transcriptional activity. (**a**) 293T17 cells were cotransfected with Vector, USP35^WT^, and USP35^S613A^ together with ERα, C3-ERE-luc reporter and TK-renilla plasmids, starved and treated with E_2_ (10 nM) for 24 h before being subjected to luciferase activity assay. TK renilla luciferase activity was used to normalize transfection efficiency. *, *p*<0.05; **, *p*<0.01. (**b**) Western blot analysis showed equal expression of USP35^WT^, USP35^S613A^ and ERα in cotransfected 293T17 cells.

Figure S9. USP35 Ser613 is important for the growth of ER+ breast cancer cells. MCF-7 and T-47D cells with Vector, USP35^WT^, USP35^S613A^ and USP35^S613E^ overexpression were subjected to colony formation assays. *, *p*<0.05; **, *p*<0.01; ***, *p*<0.001.

Figure S10. Serine613 phosphorylation does not affect deubiquitination activity of USP35 against ERα. 293T17 cells were cotransfected with HA-ubiquitin and HA-ERα plasmids together with 3×Flag-USP35^WT^ or 3×Flag-USP35^S613A^ plasmid. Cells were treated with MG132 (10 μM) for 6 h before being subjected to immunoprecipitation with anti-ERα antibody and mouse IgG (negative control).

Figure S11. AKT inhibitor does not block nuclear translocation of USP35^S613E^ in breast cancer cells. MCF-7 cells expressing vector, Flag-tagged USP35^WT^, and USP35^S613E^ were treated with vehicle or 500 nM MK2206 (AKT inhibitor) for 6 h before being immunostained with anti-Flag antibody (red) and DAPI (blue). Immunofluorescent images were captured by confocal microscopy. Bar=25 μm.

Figure S12. AKT inhibitor impairs USP35S^613E^-enhanced estrogen regulated gene expression. Indicated MCF-7 cells were pretreated with DMSO or MK2206 (100 nM) for 1 h, and then treated with vehicle or E_2_ (10 nM) for 6 h before being subjected to qRT-PCR analysis of mRNA levels of ERα target genes. *, *p*<0.05; **, *p*<0.01; ***, *p*<0.001.
